# 4‐Octyl Itaconate Alleviates Myocardial Ischemia‐Reperfusion Injury Through Promoting Angiogenesis via ERK Signaling Activation

**DOI:** 10.1002/advs.202411554

**Published:** 2025-01-21

**Authors:** Jiqin Yang, Chenqi Duan, Peng Wang, Sijia Zhang, Yuanqing Gao, Shan Lu, Yong Ji

**Affiliations:** ^1^ Key Laboratory of Cardiovascular and Cerebrovascular Medicine School of Pharmacy Nanjing Medical University 101 Longmian Avenue, Jiangning District Nanjing 211166 P. R. China; ^2^ State Key Laboratory of Frigid Zone Cardiovascular Diseases (SKLFZCD) Harbin Medical University Harbin Heilongjiang 150081 P. R. China

**Keywords:** 4‐OI, angiogenesis, IRG1, myocardial ischemia‐reperfusion injury

## Abstract

Myocardial ischemia‐reperfusion (IR) injury is a critical complication following revascularization therapy for ischemic heart disease. Itaconate, a macrophage‐derived metabolite, has been implicated in inflammation and metabolic regulation. This study investigates the protective role of itaconate derivatives against IR injury. Using a mice model of IR injury, the impact of 7‐day 4‐Octyl itaconate (4‐OI) administration on cardiac function is assessed. Exogenous administration of 4‐OI significantly reduces myocardial damage, enhances angiogenesis, and alleviates myocardial hypoxia injury during reperfusion. RNA sequencing and molecular docking techniques are used to find the target of itaconate, and changes in cardiac function are observed in Immune‐Responsive Gene1 (IRG1) global knockout mice. In cell culture studies, 4‐OI promotes endothelial cell proliferation and migration, mediated by Mitogen‐Activated Protein Kinases (MAPK) signaling pathway activation, particularly through Extracellular Signal‐Regulated Kinase (ERK) signaling. Inhibition of ERK blocks these beneficial effects on endothelial cells. Furthermore, itaconate synthesis inhibition worsens myocardial damage, which is mitigated by 4‐OI supplementation. The results indicate that 4‐OI promotes angiogenesis by activating MAPK signaling via FMS‐like tyrosine kinase 1 (Flt1), highlighting its potential as a therapeutic strategy for myocardial IR injury.

## Graphical abstract

During myocardial ischemia‐reperfusion, 4‐OI targets the intracellular domain of Flt1 to stabilize its dimer structure. The activated Flt1 promotes the phosphorylation of MAPK/ERK, leading to the activation of the MAPK/ERK signaling pathway. This subsequently facilitates the transcriptional expression of downstream angiogenesis‐related genes, promoting angiogenesis and improving reperfusion injury.

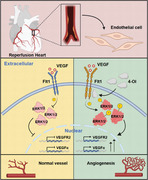

## 1 Introduction

Myocardial ischemia‐reperfusion injury (MIRI) is a prevalent complication following revascularization therapy for ischemic heart disease, resulting in complications such as arrhythmias, myocardial stunning, and microvascular obstruction. These complications exacerbate ischemic myocardial damage. Despite advancements in understanding MIRI, effective therapeutic drugs, and strategies for its treatment remain elusive. Therefore, it is of great clinical significance to further investigate the molecular mechanisms underlying MIRI and develop new therapeutic strategies. The pathogenesis of MIRI is complex, involving critical molecular processes such as mitochondrial dysfunction, oxidative stress, intracellular Ca^2+^ overload, and inflammatory responses^[^ ^1^, ^2^, ^3^, ^4^ ^]^ Ischemia‐induced tissue hypoxia and nutrient deprivation leads to energy depletion, metabolic byproduct accumulation, and impaired cellular function. Reperfusion exacerbates tissue damage through a cascade of deleterious events: the generation of reactive oxygen species (ROS) during reperfusion damages endothelial cells, induces oxidative stress, impairs endothelial function, and promotes inflammation, apoptosis, and vascular permeability. Additionally, reperfusion triggers inflammation, immune cell activation, pro‐inflammatory cytokine release, and neutrophil infiltration, further aggravating tissue damage. Calcium homeostasis disruption during reperfusion leads to mitochondrial dysfunction, apoptosis, and necrosis. Angiogenic therapy has emerged as an innovative treatment strategy to address ischemic injury via promoting vascular regeneration and tissue repair. Compared to conventional treatments such as coronary artery bypass grafting (CABG) and percutaneous coronary intervention (PCI), angiogenic approaches show the potential for fewer side effects and complications.^[^ ^5^, ^6^ ^]^ Angiogenic drugs, such as vascular endothelial growth factor (VEGF) and fibroblast growth faction (FGF), have shown promise in the treatment of ischemic heart disease. However, their clinical application is still in the trial stage due to challenges such as short half‐lives, limited targeting efficiency, and notable toxic side effects.^[^ ^6^ ^]^ With advancements in technology and research, angiogenic therapy holds great promise for becoming a key approach in treating myocardial ischemia and other ischemic diseases.

Itaconate, a natural organic compound classified as a dicarboxylic acid, is synthesized predominantly by macrophages through the Immune‐Responsive Gene 1 (IRG1), which encodes the active enzyme cis‐aconitate decarboxylase.^[^ ^7^, ^8^ ^]^ Itaconate and its derivatives can be modified to enhance specific functions and biocompatibility attracting significant attention for their potential biomedical applications.^[^ ^9^ ^]^ Itaconate has been reported to inhibit the production of inflammatory cytokines, including tumor necrosis factor‐α (TNF‐α), interleukin‐1β (IL‐1β), and interleukin‐6 (IL‐6). Also, itaconate inhibits inflammatory signaling pathways, such as nuclear factor‐κB (NF‐κB) and mitochondria‐related pathways, thereby inhibiting inflammation.^[^ ^10^, ^11^, ^12^, ^13^ ^]^ In addition, itaconate exhibits antioxidant properties, which reduce oxidative stress damage via scavenging free radicals. For instance, itaconate has been shown to regulate oxidative stress by inducing the expression of antioxidant genes through nuclear factor erythroid 2‐related factor 2 (Nrf2) activation and inhibiting the production of ROS through the downregulation of succinate dehydrogenase (SDH).^[^ ^14^ ^]^ Itaconate has been reported to target mitochondria, where it can inhibit SDH activity. This modulation of mitochondrial function could impact cellular metabolism and redox balance during MIRI.^[^ ^15^ ^]^ These pathophysiological processes play a crucial role in cardiovascular diseases such as atherosclerosis, myocardial infarction, and heart failure. As an endogenous metabolite, itaconate‐based therapeutics provide superior biocompatibility and safety compared to many exogenous drugs. Therefore, itaconate and its derivatives hold promising potential in cardiovascular research.^[^ ^16^, ^17^, ^18^ ^]^ While itaconate's anti‐inflammatory properties suggest a beneficial role in treating MIRI, further investigation is required to elucidate its specific mechanisms and efficacy in this context.^[^ ^19^, ^20^ ^]^

Here, our study aims to explore the relationship between itaconate and myocardial ischemia‐reperfusion injury. We found that 4‐Octyl itaconate (4‐OI) targets FMS‐like tyrosine kinase 1 (Flt1) during cardiac tissue reperfusion, and mediates improved endothelial function and angiogenesis. This plays a cardioprotective role. Inhibition of the Flt1 and Mitogen‐Activated Protein Kinase/Extracellular Signal‐Regulated Kinase (MAPK/ERK) signaling pathway reverses this protective effect. Additionally, IRG1‐deficient mice exhibited more severe myocardial damage, which was mitigated by 4‐OI supplementation.

## 2 Results

### 2.1 4‐OI Alleviated Myocardial Ischemia‐Reperfusion Injury

**Figure 1:**
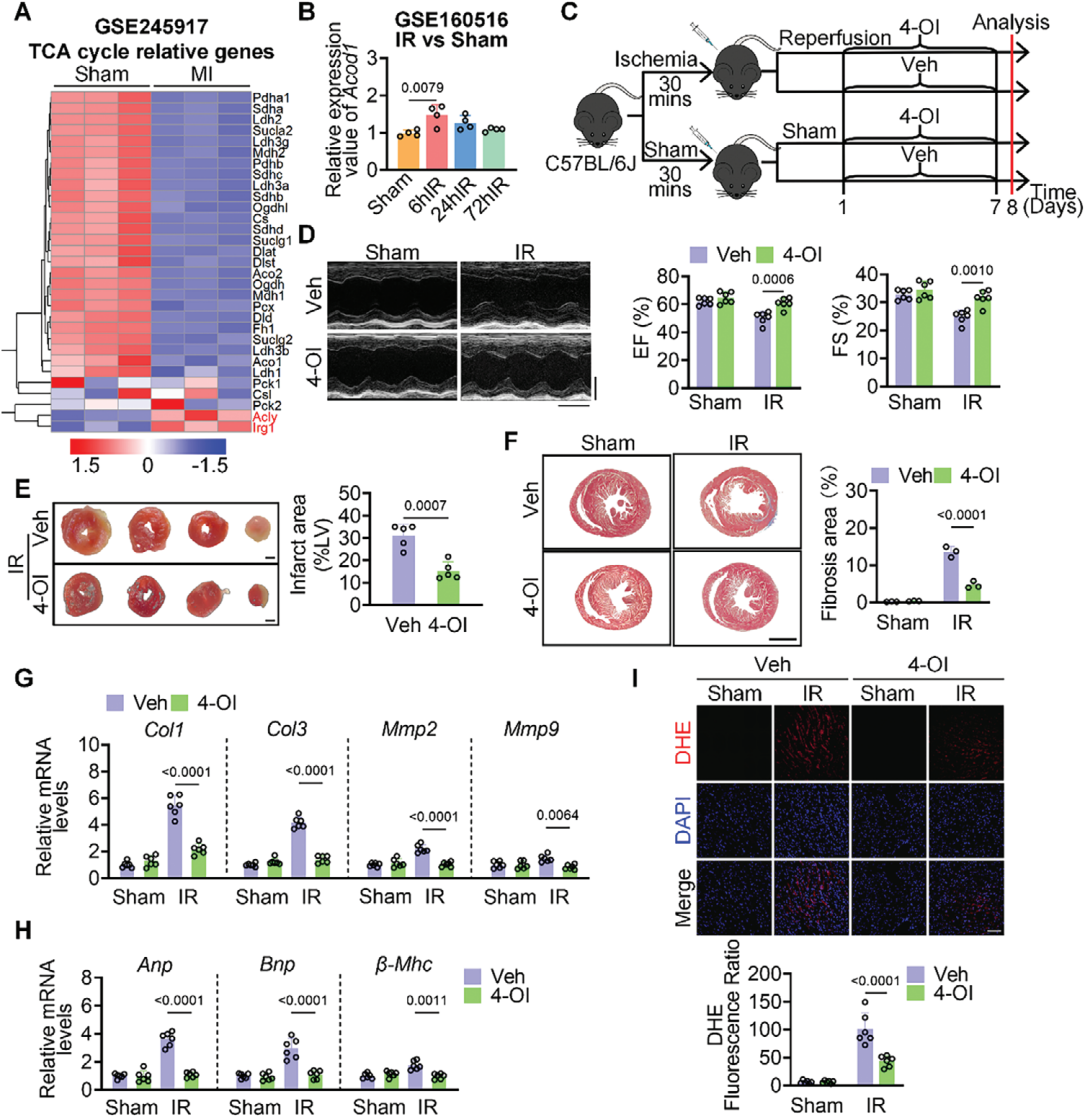
4‐OI alleviates heart dysfunction in WT mice after ischemia‐reperfusion injury. A) Heatmap plot showing the difference of TCA cycle relative gene expression in hearts in GSE245917. B) The relative expression value of Acod1 in GSE160516. C) Workflow of treatment, including IR model, administration of 4‐OI, and analysis. D) Representative echocardiograms of male mice (n = 6) from different groups, and quantification of left ventricular ejection fraction (EF, %), left ventricular fractional shortening (FS, %). E) Infarct area of I/R mice (n = 5) by TTC staining. Scale bar = 100 µm. F) Representative Masson trichrome staining from heart tissues (n = 3). Scale bar = 2 mm. G) The mRNA levels of fibrosis markers Mmp2, Mmp9, Col1, and Col3 in heart tissues (n = 6) were determined by qPCR assay. H) The mRNA levels of heart failure markers Anp, Bnp, and β‐Mhc were detected (n = 6). I) Representative images of DHE (red) staining in heart tissue (n = 6). Scale bar = 20 µm. All data are presented as mean ± SD, and *p*‐values are calculated using one‐way ANOVA with Bonferroni correction.

To identify factors that exacerbate ischemia‐reperfusion (IR) injury, we analyzed three GEO datasets, focusing first on transcriptional changes during Acute Myocardial Infarction (AMI)‐induced myocardial ischemia. In GSE245917, genes associated with the tricarboxylic (TCA) cycle were significantly down‐regulated, while immune‐responsive gene 1 (*Irg1*) and ATP‐citrate lyase (*Acly*) expression was notably upregulated during AMI (**Figure** **1**A). However, Aconitate decarboxylase 1 (*Acod1*, another manifestation of *Irg1*) expression was increased in the early stage of reperfusion and subsequently decreased over time (Figure 1B and Figure S1B, Supporting Information). Metabolomics analyses further revealed succinate accumulation during the infarction stage and its subsequent depletion during reperfusion.^[^ ^21^, ^22^ ^]^ Succinate in the reperfusion stage often exacerbates the damage by producing large amounts of reactive oxygen species. Given that itaconate inhibits succinate dehydrogenase (Figure S1A, Supporting Information), we postulate that the observed reperfusion injury, driven by succinate, may be closely linked to the expression dynamics of Irg1. We hypothesize that exogenous itaconate supplementation during reperfusion could confer cardioprotective effects. To examine this hypothesis, we initially induced an ischemia‐reperfusion (IR) model in mice, followed by consecutive intraperitoneal injections of different doses of 4‐Octyl itaconate (4‐OI) for 7 days (Figure 1C and Figure S2A, Supporting Information). A significant reduction in left ventricular ejection fraction (EF) and fractional shortening (FS) was observed in mice following the modeling of IR, whereas 10 mg kg^−1^ of 4‐OI attenuated this reduction in EF and FS values (Figure 1D). While there was no significant difference in heart size between control mice and those treated with 4‐OI, the ratios of heart weight to body weight (HW/BW) and heart weight to tibia length (HW/TL) significantly decreased in 4‐OI treated mice compared to controls following IR induction (Figure S1E, Supporting Information). Concurrently, 4‐OI reduced both the myocardial infarct area and the level of myocardial fibrosis (Figure 1E–G). The long‐term reperfusion process resulted in increased expression of heart failure biomarkers, including atrial natriuretic peptide (*Anp*), brain natriuretic peptide (*Bnp*), and β‐myosin heavy chain (*β‐Mhc*), while 4‐OI inhibited their expression (Figure 1H). In addition, reduced levels of reactive oxygen species (ROS) were detected in myocardial tissue after the modeling process (Figure 1I). Compared with the IR group, 4‐OI significantly reduced TUNEL‐positive signaling and Caspase3 activity in myocardial tissue (Figure S3A,B, Supporting Information). This was accompanied by a decrease in apoptosis‐related proteins, including Bcl2‐associated X (Bax) and B‐cell lymphoma‐2 (Bcl2) (Figure S3C, Supporting Information). Moreover, no sex difference was detected in this protective effect (Figure S2B, Supporting Information).

### 2.2 4‐OI Promotes Angiogenesis in the Reperfusion Phase

**Figure 2:**
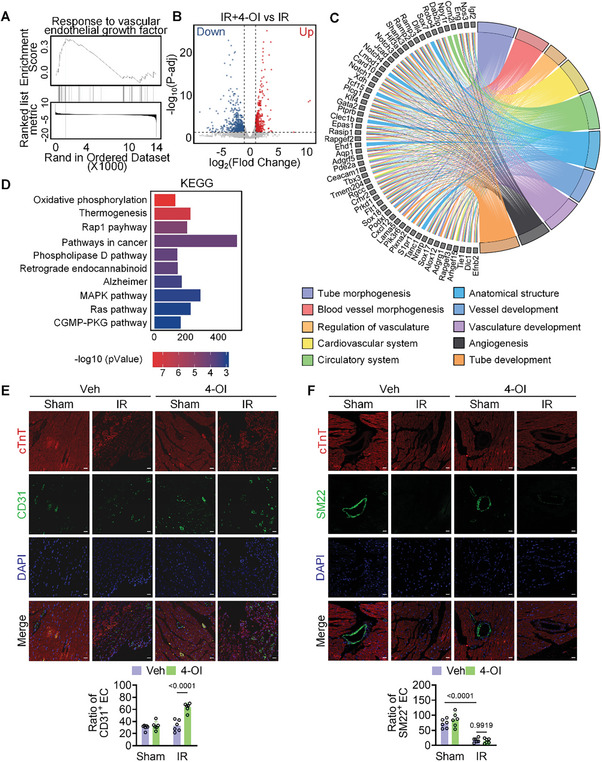
The protective effect of 4‐OI on cardiac function is related to angiogenesis. A) GSEA enrichment plot of Response to vascular endothelial growth factor in 4‐OI treatment classified analysis. B) Volcano plot showing the difference of gene expression in hearts between WT mice treated veh and treated 4‐OI subjected to IR operation. C) The top10 GO enrichment analysis of upregulated genes after treating 4‐OI subjected to IR operation. D) The top 10 KEGG pathway analysis of a total of these 416 genes. E) Immunofluorescent staining was performed with antibodies against CD31 (green) and cardiomyocytic marker cTnT (red) (n = 6). Scale bar = 20 µm. F) Immunofluorescent staining was performed with antibodies against SM22 (green) and cardiomyocytic marker cTnT (red) (n = 6). Scale bar = 20 µm. All data are presented as mean ± SD, and the P‐values are calculated using one‐way ANOVA with Bonferroni correction.

Then, we investigated how exogenous administration improved IR injury by employing RNA‐seq analysis. RNA‐seq was performed on myocardial tissues from the ischemic border zone of three different groups. Separate Gene Set Enrichment Analysis on the three groups revealed that the vehicle‐treated IR (IR) group, as opposed to the sham surgery (Sham) group, was mainly associated with processes related to the extracellular matrix and cell adhesion pathways (Figure S4C, Supporting Information). In contrast, the 4‐OI‐treated IR (4‐OI) group exhibited a predominant association with the regulation of vascular endothelial growth factor compared to the IR group (**Figure** **2**A). After 7 days of IR injury, the 4‐OI group showed up‐regulation of 419 genes with down‐regulation of 626 genes compared to the IR group (Figure 2B). To investigate the specific mechanisms underlying the alleviation of myocardial damage by administration, we focused on the biological processes associated with the 419 upregulated genes. Gene ontology (GO) and Kyoto Encyclopedia of Genes and Genomes (KEGG) enrichment analysis were performed on these genes, and showed that these genes were mainly enriched in biological processes such as angiogenesis (Figure 2C,D). Subsequent immunofluorescence staining showed significantly increased expression of the endothelial cell marker CD31 in ischemic and border areas compared to the SM22 smooth muscle cell marker (Figure 2E,F). These findings suggest that treatment with 4‐OI may contribute to the repair of myocardial injury during reperfusion by modulating angiogenesis.

### 2.3 The Ability of 4‐OI to Induce Angiogenesis Is Related to Flt1

**Figure 3:**
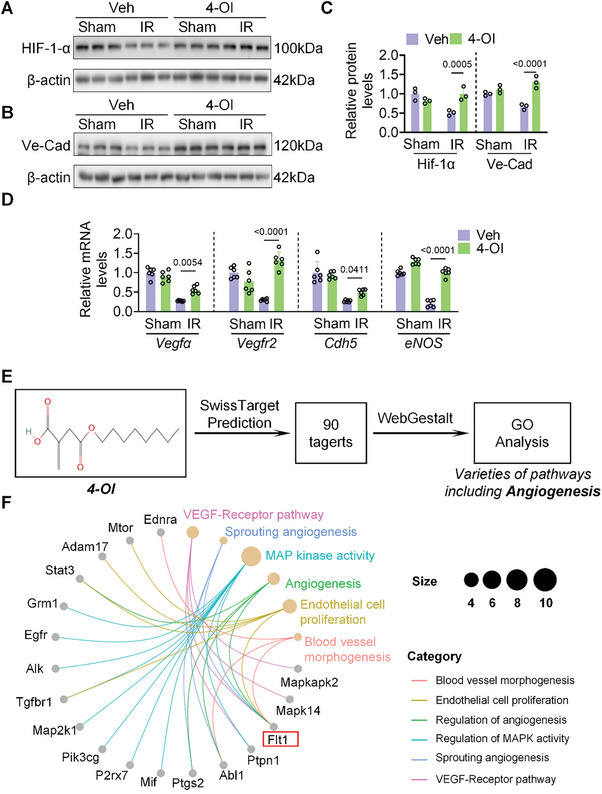
4‐OI can significantly promote angiogenesis in ischemic hearts. A–C) The protein levels of HIF‐1‐α and VE‐Cad in heart tissues (n = 3) after 4‐OI administration. D) The mRNA levels of Vegfα, Vegfr2, Cdh5, and eNOS in heart tissues (n = 6) from WT with Veh or 4‐OI administration. E) The brief flow chart for 4‐OI target prediction. F) The representative GO analysis of the predicted target. All data are presented as mean ± SD, and *p*‐values are calculated using one‐way ANOVA with Bonferroni correction.

Based on prior findings that endothelial cell proliferation was a critical step in blood vessel development, our analysis of graphene oxide enrichment indicated that continuous administration promoted angiogenesis during prolonged reperfusion. We found hypoxia inducer 1‐α (HIF1‐α) and vascular endothelial cadherin (VE‐cad), both crucial for endothelial cell growth, were significantly suppressed following IR. After administration of 4‐OI, the expression of these two proteins was significantly increased (**Figure** **3**A–C). The mRNA levels of growth factor receptor, ligand, and endothelial cell proliferation marker genes were significantly reduced after IR. Conversely, administration of 4‐OI led to a significant increase in mRNA levels of these genes (Figure 3D). Subsequently, the SwissTarget target prediction website (http://www.swisstargetprediction.ch/) was utilized to predict the targets of 4‐OI. Ninety candidate target genes and the biological processes involved were enriched (Figure 3D). Multiple targets related to the regulation of angiogenesis and MAP kinase activity were found, matching the RNA sequencing results. Among these targets, FMS‐like tyrosine kinase 1 (Flt1) emerged as a key player involved in all angiogenesis processes (Figure 3E). The molecular Operating Environment (MOE) software was used to confirm the interaction between 4‐OI and Flt1 (Figure S6A, Supporting Information). The Cellular Thermal Shift Assay (CETSA) assay also confirmed the results of our molecular dynamics simulation, showing that binding of 4‐OI to Flt1 significantly improved the thermal stability of the protein (Figure S6B, Supporting Information).

### 2.4 4‐OI Improved Cell Migration in Human Umbilical Vein Endothelial Cells (HUVECs)

**Figure 4:**
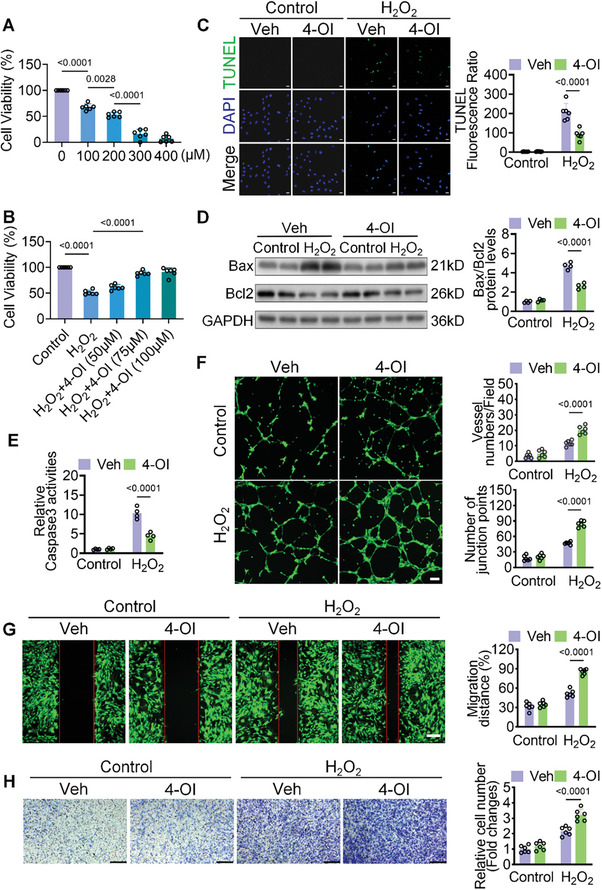
4‐OI mitigates cell injury in HUVECs under hypoxic conditions. A) The viability of HUVEC cells (n = 6) after treatment with various concentrations of H_2_O_2_ (0, 100, 200, 300, 400 µm) for 12 h, as detected by the CCK8 assay (n = 6). B) The viability of cells after H_2_O_2_ stimulation (200 µm) for 6 h, followed by treatment with different doses (0, 50, 75, 100 µm) of 4‐OI for 12 h, as analyzed by the CCK8 assay (n = 6). C,D, The HUVEC cells were treated with 200 µm H_2_O_2_ or a combination of 75 µm and 200 µm H_2_O_2_. C) Representative images of TUNEL (green) staining in HUVEC cells (n = 6). Scale bar = 20 µm. D) The expression of Bax and Bcl2 at protein levels was detected in HUVEC cells (n = 4). E) The relative Caspase 3 activities in hearts from different groups were detected (n = 4). F–H) The HUVEC cells were treated with 50 µm H_2_O_2_ or a combination of 75 µm and 50 µm H_2_O_2_. F) The number of tubes and junction points in tube formation (n = 6). Scale bar = 100 µm. G) The migration distance of HUVECs treated with 4‐OI was measured by the wound healing assay (n = 6). Scale bar = 200 µm. H) 4‐OI‐induced endothelial migration using the transwell migration assay (n = 6). Scale bar = 100 µm. All data are presented as mean ± SD, and *p*‐values are calculated using one‐way ANOVA with Bonferroni correction.

Human umbilical vein endothelial cells (HUVECs) were exposed to H_2_O_2_ to simulate hypoxic injury in myocardial endothelial cells, followed by observation under control solvent or 4‐OI treatment conditions. Initially, HUVECs were exposed to gradient concentrations of H_2_O_2_ for 6 h, followed by cytotoxicity assessment using the Cell Counting Kit‐8 (CCK8) method. As depicted illustrated in **Figure** **4**A, exposure to H_2_O_2_ resulted in a dose‐dependent decrease in HUVECs cell viability. Compared with the control group, the viability of HUVECs reached 56.30% at 200 µm. Hence, 200 µm H_2_O_2_ was used to set up cell damage models. To evaluate whether 4‐OI has an inhibitory effect on H_2_O_2_‐induced cell death, we analyzed cell viability. CCK8 experiments showed that 4‐OI significantly increased cell viability in a dose‐dependent manner. Cell death was significantly inhibited under 75 µM 4‐OI treatment (Figure 4B). 4‐OI treatment markedly attenuated hypoxia‐induced apoptosis in endothelial cells. As shown by a reduction in TUNEL‐positive signals, a lower Bax/Bcl2 protein ratio, and decreased Caspase‐3 activity (Figure 4C–E). Furthermore, low‐dose H_2_O_2_ (50 µm) treatment promoted the angiogenic capacity of HUVECs, which was further augmented by 4‐OI. This was evidenced by an increased number of blood vessels and junction points (Figure 4F). Scratch and transwell assays demonstrated that low‐dose H_2_O_2_ treatment improved HUVECs migration, which was further amplified by 4‐OI treatment (Figures 4G,H). Collectively, these findings indicate that 4‐OI treatment enhances endothelial cell viability and potentiates angiogenesis at the cellular level.

### 2.5 4‐OI Enhanced the Activity of Flt1 to Activate the MAPK/ERK Signaling Pathway

**Figure 5:**
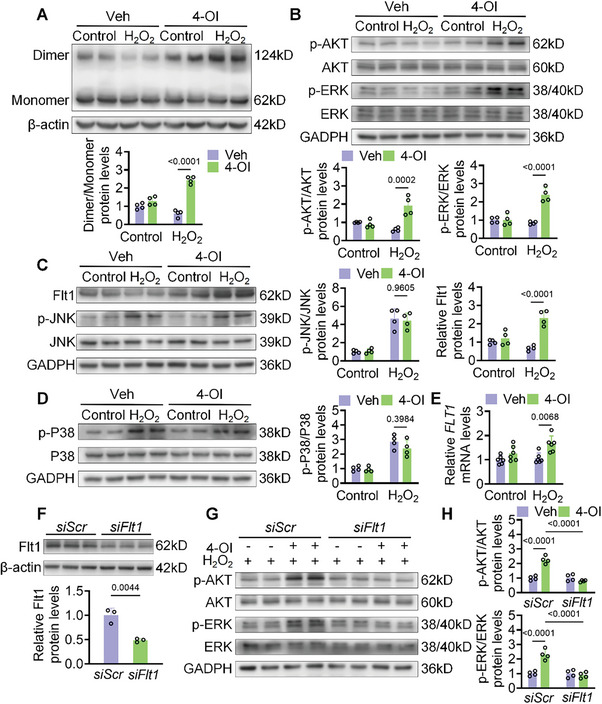
4‐OI activates MAPK/ERK signaling pathway by targeting Flt1. A–E) The HUVEC cells were treated with 200 µm H_2_O_2_ or a combination of 75 µm 4‐OI and 200 µm H_2_O_2_. A) Immunoblot analysis of Flt1 oligomerization in HUVECs. B) The expression of ERK and p‐ERK or AKT and p‐AKT at protein levels were detected in HUVEC cells (n = 4). C) The expression of Flt1 or JNK and p‐JNK at protein levels was detected in HUVEC cells (n = 4). D) The expression of P38 and p‐P38 at protein levels were detected in HUVEC cells (n = 4). E) Under hypoxia condition, the mRNA level of Flt1 was detected in HUVEC cells treated with or without 4‐OI. F) The protein level of Flt1 was detected in HUVEC cells treated with siScr or siFlt1 (n = 3). G,H) Under hypoxia condition, the HUVEC cells were treated with siScr or siFlt1. G) The representative image of Western blot assay. H) The expression of p‐ERK/ERK or p‐AKT/AKT at protein levels was detected in HUVEC cells (n = 4). All data are presented as mean ± SD, and *p*‐values are calculated using one‐way ANOVA with Bonferroni correction.

Itaconate is a metabolite that inhibits the activity of pyruvate kinase (PK) and succinate dehydrogenase (SDH) by altering their catalytic efficiency and metabolic pathways. Previous results showed that itaconate derivative 4‐OI interacts with Flt1. Activation of downstream signaling pathways by Flt1 requires ligand‐receptor binding, followed by the formation of a dimer structure. Molecular dynamics simulations showed that 4‐OI can bind to the intracellular domain of Flt1 (Figure S6A, Supporting Information), suggesting that 4‐OI may enhance the activity of Flt1 by stabilizing its dimer structure. We found that H_2_O_2_ treatment decreased the dimer structure of Flt1 in HUVECs, while 4‐OI treatment significantly increased the dimer structure of Flt1 (**Figure** **5**A). Studies have shown that 4‐OI activates Mitogen‐Activated Protein Kinase/Extracellular Signal‐Regulated Kinase (MAPK/ERK) signaling pathways in human hepatocellular carcinoma cells.^[^ ^23^ ^]^ Flt1 is an upstream regulator of the MAPK/ERK pathway. We aimed to determine whether the activation of Flt1 in HUVECs affects this pathway. As shown, 4‐OI promotes the phosphorylation of ERK in endothelial cells, but has no significant effect on the phosphorylation activation of other members of MAPK (Figure 5B–D). This result suggests that 4‐OI can stabilize the dimer form of Flt1 and activate the MAPK/ERK signaling pathway, subsequently promoting the proliferation and migration of endothelial cells. Activation of this pathway is usually accompanied by transcriptional activation. Therefore, we hypothesized that both the activity and expression levels of Flt1 were altered. As suspected, 4‐OI treatment increased mRNA and protein levels of Flt1. And the protein levels were found it be not the same as mRNA levels (Figure 5C,D). Given that 4‐OI treatment led to increased phosphorylation of AKT in endothelial cells (Figure 5B), which has been reported to be associated with protein ubiquitination binding,^[^ ^24^, ^25^ ^]^ we speculated that 4‐OI affects Flt1 ubiquitination and degradation. Our observations indicated that inhibitors of protein synthesis decreased Flt1 protein expression in a time‐dependent manner (Figure S7A, Supporting Information), whereas the administration of protease inhibitors reversed this reduction. Immunoprecipitation assays further confirmed that 4‐OI reduced Flt1 ubiquitination levels (Figure S7B). To further verify the key role of Flt1. Small Interfering RNA (siRNA) was used to reduce the expression of Flt1 in HUVEC cells (Figure 5F). The results suggest that Flt1 knockdown can reverse 4‐OI‐induced phosphorylation of MAPK/ERK and AKT (Figure 5G). Similarly, angiogenesis and endothelial cell migration were significantly inhibited (Figure S8A–C, Supporting Information).

### 2.6 Inhibition of MAPK/ERK Can Reverse the Effect of 4‐OI

**Figure 6:**
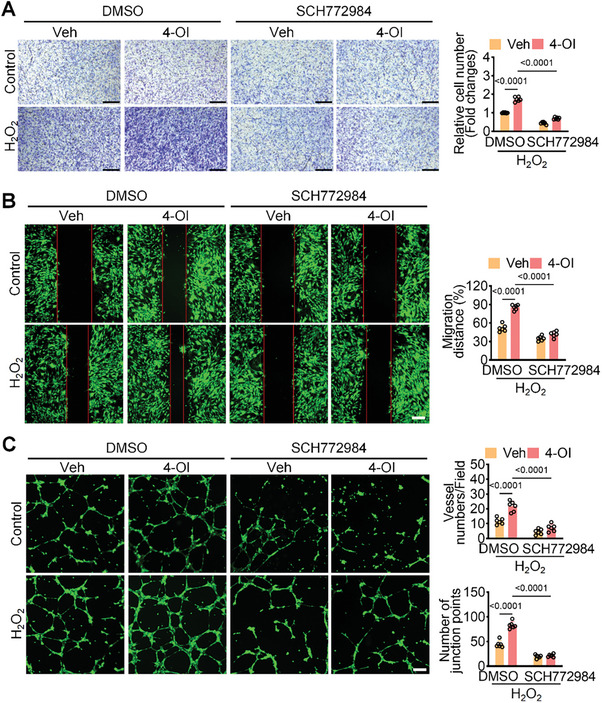
Inhibition of ERK1/2 reverses the protective effect of 4‐OI on HUVECs. A) Transwell migration assay showed that SCH772984 inhibited endothelial cell migration induced by 4‐OI (n = 6). Scale bar = 200 µm. B) Wound healing assay for detecting the migratory ability of DMSO and SCH772984 treated with 4‐OI under hypoxic condition (n = 6). Scale bar = 100 µm. C) The SCH772984 fails to promote tube formation in HUVECs under hypoxic condition (n = 6). Scale bar = 100 µm. All data are presented as mean ± and SD, and *p*‐values are calculated using one‐way ANOVA with Bonferroni correction.

To confirm the role of 4‐OI in promoting endothelial cell proliferation and migration through the activation of MAPK signaling pathways targeting Flt1, we employed SCH772984, a specific inhibitor of ERK, to inhibit its activity in HUVECs. The results indicated that SCH772984 had no obvious impact on the migration and angiogenesis of untreated HUVECs. Under H_2_O_2_ treatment, SCH772984 decreased the migration ability of the untreated group and the number of formed blood vessels. However, in the 4‐OI treated group, SCH772984 markedly reversed the enhancement of cell migration and angiogenesis induced by 4‐OI (**Figure** **6**A–C). These findings suggest that 4‐OI promotes cell migration and angiogenesis by targeting Flt1 and activating the MAPK signaling pathways. Based on these findings, we delineated a pathway whereby exogenous administration of 4‐OI, a derivative of itaconate, is implicated in endothelial cell proliferation and migration, angiogenesis promotion, and mitigation of myocardial damage during long‐term reperfusion.

### 2.7 Blocking the Endogenous Itaconate Synthesis Aggravated Myocardial Ischemia‐Reperfusion Injury

**Figure 7:**
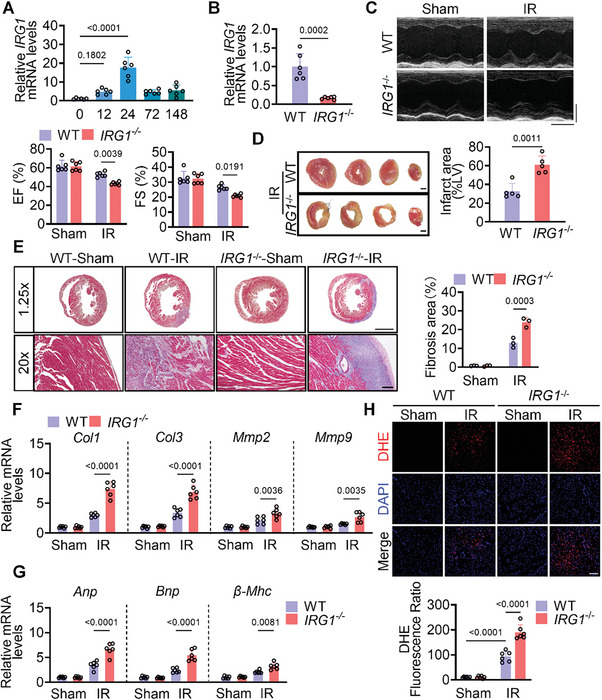
IRG1 deficiency aggravated heart dysfunction after IR injury. A) The mRNA levels of IRG1 in cardiac tissues (n = 6) from mice were monitored by qRT‐PCR assay. B) The mRNA levels of IRG1 in cardiac tissues (n = 6) of C57BL/6 (WT) or *IRG1* ^‐/‐^ mice. C) Representative echocardiograms of mice (n = 6) from different groups, and quantification of left ventricular ejection fraction (EF, %), left ventricular fractional shortening (FS, %). D) Infarct area of IR mice (n = 5) by TTC staining. Scale bar = 100 µm. E) Representative Masson trichrome staining from heart tissues (n = 3). The scale bar of 1.25x = 2 mm. The scale bar of 20x = 100 µm. F) The mRNA levels of fibrosis markers Mmp2, Mmp9, Col1, and Col3 in heart tissues (n = 6) were determined by qPCR assay. G) The mRNA levels of heart failure markers Anp, Bnp, and β‐Mhc were detected (n = 6). H) Representative images of DHE (red) staining in heart tissue (n = 6). Scale bar = 20 µm. All data are presented as mean ± and SD, *p*‐values are calculated using one‐way ANOVA with Bonferroni correction.

**Figure 8:**
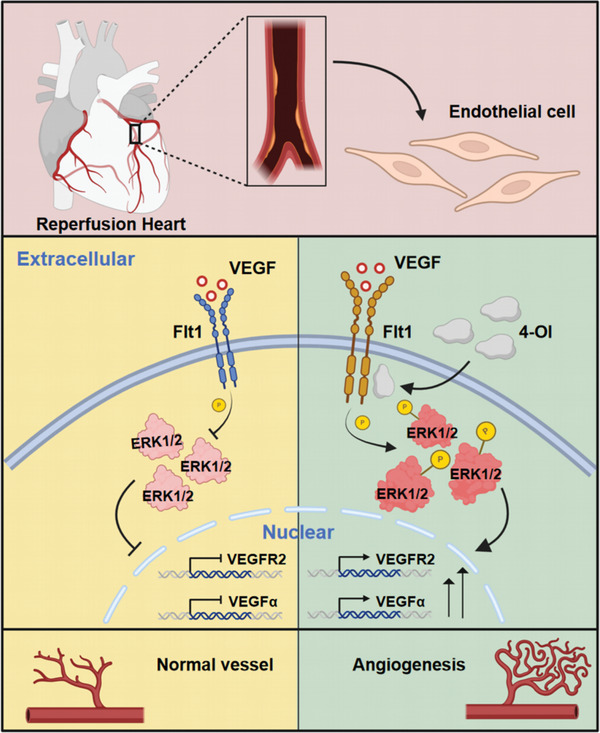
The schematic diagram of the main molecular pathways.

Our prior data has shown the exogenous 4‐OI could protect the heart from IR injury. The enzyme Irg1, which is crucial for endogenous itaconate synthesis, exhibited a substantial increase in mRNA levels during the early reperfusion phase, followed by a return to near‐baseline levels during the middle and late reperfusion phases (**Figure** **7**A). To examine the role of endogenous itaconate during prolonged reperfusion, CRISPR‐Cas9 was employed to global knockout IRG1 (Figure S9A, Supporting Information, and Figure 7B). Then, we conducted myocardial reperfusion experiments on wild‐type (WT) and *IRG1^‐/^* ^‐^ mice over a 7‐day period. Echocardiography revealed that IRG1 deficiency exacerbated the pronounced reduction in EF and FS post‐reperfusion, indicating worsened cardiac dysfunction in the absence of Irg1 (Figure 7C). While there was no notable difference in heart size between WT and *IRG1^‐/‐^* mice, the ratios of heart weight to body weight (HW/BW) and heart weight to tibia length (HW/TL) were significantly elevated in *IRG1* ^‐/‐^ mice post‐reperfusion compared to WT mice (Figure S9D, Supporting Information). The absence of Irg1 significantly increased myocardial infarction size and the level of myocardial fibrosis (Figure 7D–F). The expression of biomarkers of heart failure was increased, including atrial natriuretic peptide (*Anp*), brain natriuretic peptide (*Bnp*), and β‐myosin heavy chain (*β‐Mhc*). Moreover, the levels of ROS in myocardial tissue were elevated (Figure 7G,H). Compared with wild‐type mice, the TUNEL‐positive signal, Caspase3 activity, and the expression of apoptotic markers Bax and Bcl2 in the heart tissue of *IRG1*‐deficient mice were further increased (Figure S10A–C, Supporting Information). This indicated that IRG1 deficiency promoted apoptosis induced by IR. Subsequently, exogenous 4‐OI was subsequently administered to the knockout mice, resulting in a significant restoration of cardiac function, as evidenced by increased EF and FS values, along with alleviated myocardial fibrosis and a substantial reduction in levels of ROS in myocardial tissue (Figure S11A–C, Supporting Information). These findings suggest that blocking endogenous itaconate anabolism could exacerbate myocardial injury during reperfusion, whereas administration of 4‐OI mitigated such. We observed that the increase of Irg1 in the early stage of reperfusion usually came from the infiltration of macrophages (Figure S12A, Supporting Information). Isolated *IRG1*‐deficient endothelial cells did not cause changes in angiogenesis and cell migration ability, and exogenous 4‐OI supplementation had the same improvement ability for wild‐type and defective endothelial cells (Figure S12B,C, Supporting Information). However, the heart injury caused by IRG1 deficiency seems to be related to the cross‐linking between macrophages and endothelial cells. Necrotic myocardial cell supernatants (NMCS) treatment bone marrow‐derived macrophages (BMDMs) were used to simulate the inflammatory state in the early stages of reperfusion. Wild‐type BMDMs supernatant showed no significant changes in endothelial cell angiogenesis and cell migration, but *IRG1*‐deficient BMDMs supernatant significantly impaired endothelial function. Supplementation with 4‐OI significantly promotes angiogenesis and cell migration (Figures S12D,E, Supporting Information). These results suggest that 4‐OI can directly target endothelial cells independently of endogenous itaconic acid. In general, 4‐OI targets Flt1 to stabilize the dimer structure and phosphorylates to activate MAPK/ERK and AKT. Transcription activates the expression of genes associated with angiogenesis and promotes angiogenesis (**Figure** **8**).

## 3 Discussion

In this study, we observed increased levels of ROS production and apoptosis in myocardial tissue during the late reperfusion period. Sustained 4‐Octyl itaconate (4‐OI) administration effectively mitigated these adverse changes. This study demonstrates, for the first time, that the 4‐OI targets the receptor protein Flt1, thereby activating the MAPK signaling pathway and enhancing Extracellular Signal‐Regulated Kinase1/2 (ERK1/2) phosphorylation. This cascade promotes endothelial cell proliferation and migration, induces angiogenesis in reperfused myocardium, and exerts a cardioprotective effect. Specifically, we found that 4‐OI markedly attenuates the H_2_O_2_‐induced decline in HUVECs activity while enhancing its angiogenic potential; this effect is reversed upon Flt1 knockdown and ERK1/2 inhibition. Furthermore, deficiency of Immune Response Gene 1 (IRG1), a key enzyme in itaconate synthesis, exacerbates cardiac damage and fibrosis post‐ischemia reperfusion (IR). Notably, supplementation of 4‐OI can ameliorate the IR damage associated with IRG1 deficiency.

During ischemic injury, early restoration of blood flow can facilitate the recovery of ischemic organs; however, it can also induce irreversible damage, termed ischemia‐reperfusion injury (IRI). IRI is characterized by oxidative stress, inflammation, apoptosis, and fibrillation. In the later stage of IRI, excessive ROS accumulation in mitochondria directly damages mitochondrial components and exacerbates oxidative stress, resulting in decreased mitochondrial membrane potential (MMP), cytochrome c release, increased ROS production, and impaired ATP synthesis.^[^ ^26^, ^27^, ^28^, ^29^ ^]^ Sustained mitochondrial damage further activates apoptotic signals by altering the expression of Bcl‐2 family proteins, subsequently disrupting cell homeostasis.^[^ ^30^, ^31^, ^32^ ^]^ Itaconate, as an endogenous metabolite, has exhibited a protective effect on various pathologies by modulating oxidative stress and inflammatory responses.^[^ ^10^ ^]^ Studies have demonstrated that knockdown of itaconate synthetase Irg1 exacerbates liver injury post‐IRI in mice, evidenced by elevated serum alanine aminotransferase (ALT) levels and histological analysis, whereas 4‐OI treatment reduced hepatocyte apoptosis post‐IRI. Itaconate ameliorates brain and heart IRI^[^ ^33^ ^]^: in the brain, itaconate elevates brain glutathione levels, diminishes levels of ROS and Nitrogen Species (RNS), enhances hemodynamics, and mitigates leukocyte adhesion, consequently enhancing nerve function.^[^ ^15^ ^]^ Additionally, 4‐OI protects neuronal cells from H_2_O_2_‐induced oxidative damage by activating the Nrf2 signaling pathway.^[^ ^34^ ^]^ In cardiac IRI, itaconate modulates tricarboxylic acid (TCA) cycle remodeling and macrophage activation by inhibiting succinate dehydrogenase (SDH), regulating the production of inflammatory cytokines, electron transport chain flow, as well as ROS production.^[^ ^35^ ^]^

In our study, GEO datasets were re‐analyzed to investigate metabolic changes during myocardial ischemia. Ischemia reduces or interrupts blood flow to cardiomyocytes, resulting in hypoxia and impaired TCA cycle which causes energy metabolism disorder and tissue l damage. The metabolic imbalance and cellular damage during ischemia predispose the myocardium to further injury during reperfusion, as the TCA cycle is a critical pathway for cellular energy production. We first analyzed the expression levels of TCA cycle‐related genes in GSE245917 dataset. Notably, only *Irg1* and *Acly* were significantly increased during myocardial ischemia. Further analysis of GSE160516 and GSE193997 revealed that *Irg1* expression dynamically in different stages of IR. It was significantly increased in the early stages of reperfusion, and gradually returned to the baseline levels. Interestingly, succinate levels also exhibited dynamical changes during IRI.^[^ ^21^ ^]^ Given itaconate's inhibitory effect on succinate dehydrogenase, we hypothesized that it may have a protective effect similar to malonate in alleviating reperfusion injury.^[^ ^36^ ^]^ To investigate this, we explored the role of the itaconate derivative 4‐OI in long‐term IR injury. Mice subjected to IR injury were intraperitoneally injected with solvents or different doses of 4‐OI for 7 days. The results further validated our hypothesis that 4‐OI is effective in treating the IRI.

To elucidate the downstream pathway, transcriptomic analysis highlighted biological processes associated with angiogenesis and cell migration. Immunofluorescence showed that 4‐OI specifically promoted endothelial cell proliferation but not smooth muscle cells. Subsequent western blot and qRT‐PCR analysis confirmed increased expression of vascular endothelial marker genes. Target prediction and molecular docking analysis suggested that 4‐OI binds to the intracellular domain of Flt1, a receptor tyrosine kinase essential for angiogenesis.^[^ ^1^, ^8^, ^11^, ^14^, ^37^ ^]^ Itaconate and its derivatives, including 4‐OI, are known to act as competitive inhibitors or allosteric regulators, modifying enzyme activity through conformational changes. Using CESTA and Western Blot assays, we verified that 4‐OI stabilized Flt1 dimerization and enhanced its activity. Flt1 activation by 4‐OI influenced multiple signaling pathways, with a focus on MAPK/ERK signaling based on previous studies and GO enrichment analysis.^[^ ^23^ ^]^ This pathway plays a key role in cell proliferation, differentiation, and survival, critical for endothelial cell migration, proliferation, and tube formation.^[^ ^38^, ^39^, ^40^, ^41^ ^]^ 4‐OI treatment increased ERK1/2 and AKT phosphorylation, stabilized Flt1 expression under hypoxic conditions by reducing its ubiquitination, and enhanced angiogenesis. Notably, ERK1/2 inhibition abrogated 4‐OI‐induced angiogenesis, highlighting its essential role in this process.

Reperfusion can cause additional tissue damage through mechanisms such as oxidative stress, inflammation, and metabolic disorders. Macrophage polarization and metabolic status play a dual role in inflammatory response and tissue repair, with itaconate acting as a key regulator.^[^ ^42^, ^43^, ^44^ ^]^ Using global *IRG1* knockout mice, we observed worsened reperfusion injury, characterized by cardiac dysfunction and fibrosis, highlighting the protective role of endogenous itaconate. Supplementation with 4‐OI reversed these dysfunctions, demonstrating its therapeutic potential. Endothelial cells from *IRG1*‐defecient and WT mice showed similar function, and the 4‐OI improved endothelial performance in both cases. Macrophages and endothelial cells interact dynamically in the cardiac microenvironment. Moderately M1‐polarized macrophages promote angiogenesis, whereas macrophages with excessive M1‐polarization seriously damage endothelial cells.^[^ ^45^, ^46^ ^]^ Itaconate could shift M1‐polarized macrophages toward an M2‐like phenotype, mitigating such damage. Bone marrow‐derived macrophage (BMDMs) from *Irg1*‐deficient mice treated with necrotic myocardial cell supernatants impaired endothelial function, reducing angiogenesis and migration.^[^ ^47^ ^]^ 4‐OI restored these functions, indicating that itaconate directly targets endothelial cells. As reperfusion progressed, *Irg1* expression decreased to baseline, while 4‐OI supplementation could promote angiogenesis, alleviate oxidative stress damage, improve blood flow supply, and relieve cardiomyocyte stress, underscoring its therapeutic promise in myocardial IR.

Our study revealed a key breakthrough: 4‐OI administration during reperfusion targets Flt1, activates the MAPK/ERK signaling pathway, and enhances endothelial cell function. These findings reveal the molecular mechanism of 4‐OI's angiogenic effects and highlight its potential application in the treatment of myocardial ischemia‐reperfusion injury. As a derivative of endogenous metabolites, 4‐OI offers advantages over traditional angiogenic drugs, including lower toxicity, higher modifiability, and greater availability.^[^ ^48^, ^49^ ^]^ Yet it remains a problem of lacking an efficient drug delivery system, hydrogel could further enhance its therapeutic potential by enabling targeted delivery, optimizing dosage, and improving treatment timing.^[^ ^50^, ^51^ ^]^ In conclusion, our findings provide a new perspective for 4‐OI treatment of myocardial ischemia‐reperfusion injury and other cardiovascular diseases.

## 4 Experimental Section

### Animals

Male mice (7‐8 weeks old, 22–25 g) were used in this study. IRG1^‐/‐^ mice (Gempharmatech Co., Ltd, Nanjing, China) were treated with CRISPR‐Cas9 to obtain global knockout mice, which were bred in the laboratory. All mice were maintained in a temperature‐controlled facility with 12 h light/ dark cycle at 23 ± 3 °C and 30–70% humidity. All animal experiments were approved by the Ethic Committees of Nanjing Medical University and in accordance with the Guide for the Care and Use of Laboratory in Nanjing Medical University (Ethical Approval Number: 221 207). The experimenters were blind to treatment/genotype grouping information during the experiment and quantification.

4‐Octyl itaconate (10 mg kg^−1^ d^−1^, MedChemExpress, USA) was administrated after surgery in C57BL/6 mice for 7 days. The saline solution was the vehicle as control.

### Mouse Model of Myocardial Ischemia‐Reperfusion Injury

Cardiac IR injury was induced by 30 min ischemia, followed by 24 h reperfusion. Briefly, male mice were anesthetized with 1–2% isoflurane (RWD Life Science). The anesthetized mice were intubated and ventilated using a rodent ventilator with a tidal volume of 200 µl and a frequency of 110 breaths per minute (R415; RWD Life Science, China). Then, the skin surface of the left chest was disinfected and a thoracotomy through 3, 4 intercostal areas was performed to expose the heart. The left anterior descending coronary artery (LAD) was occluded by tying a slipknot with a 7‐0 silk suture 1–2 mm from the lower edge of the left atrium. After 30 min, the slipknot was released to reperfusion. For sham group mice, the operation followed the same procedure without ligation.

### Generation of Bone Marrow‐Derived Macrophages

Bone marrow‐derived macrophages (BMDMs) were isolated from the femurs of adult mice and cultured in complete 1640 medium (10% fetal bovine serum, 1% penicillin‐streptomycin) supplemented with 40 ng mL^−1^ M‐CSF (HY‐P7050, MCE) for 5–7 days. The medium was changed on days 3, 5, and 7 with half‐volume replacement. The bone marrow cells were then differentiated into BMDMs for subsequent experiments.

### Isolation of Mouse Kidney Endothelial Cells

Mouse kidney endothelial cells (MKECs) were isolated from the kidneys of 8–10‐week‐old adult mice. Briefly, anti‐mouse ICAM‐2 Dynabeads were prepared by incubating Dynabeads M‐450 Sheep anti‐Rat IgG (11 035, Thermo Fisher) with ICAM2 antibody (553 325, Pharmingen). After euthanizing the mouse, the kidney tissue was soaked in 70% ethanol for disinfection, and the kidney was harvested. The dissociated tissue was incubated in collagenase I (LS004194, Worthington) for 45 min, followed by overnight incubation with anti‐mouse ICAM‐2 Dynabeads. The MKECs were then isolated using ICAM‐2 Dynabeads for secondary separation.

### Preparation and Application of Necrotic Myocardial Cell Supernatant

Necrotic myocardial cell supernatant (NMCS) was prepared based on previous reports. Briefly, freshly excised hearts from 8–12‐week‐old C57BL/6 mice were rinsed in cold PBS and then minced into small pieces in cold PBS. The minced tissue was resuspended in PBS and subjected to three freeze‐thaw cycles (10 min on dry ice with methanol (<−80 °C), followed by 3 min in a 37 °C water bath). Afterward, the suspension was centrifuged at 12 000 × *g* for 10 min. The supernatant collected after centrifugation was diluted 1:1 with culture medium and used to stimulate BMDMs

### Bioinformatic Analysis

RNA expression data in myocardial infarction (GEO: GSE245917) and myocardial ischemia‐reperfusion injury (GEO: GSE160516, GSE193997) was obtained from the NCBI GEO database. The clean reads were aligned to mm10 by Hisat2 (v2.1.0). DESeq2 software package (version 1.30.1) was used to screen differentially expressed genes (fold change >2 and *p*‐value < 0.05). The heatmap was generated with https://www.bioinformatics.com.cn, an online bioinformatic analysis platform.

### Echocardiography

The murine cardiac M‐mode echocardiography was acquired using the Vevo2100 imaging system (VisualSonics, Toronto, Canada) to assess mouse cardiac function. Briefly, following the removal of chest fur with a surgical blade, the mice were coated with a medical ultrasound coupling agent (Tianjin Yajie Medical Materials Co., Ltd., Tianjin, China). Two‐dimensional M‐mode traces were recorded on short‐axis sections (at the level of the papillary muscles) and long‐axis sections (below the level of the papillary muscles) adjacent to the sternum. M‐mode echocardiographic data covering at least six consecutive cardiac cycles were collected. Subsequently, numerical values for left ventricular end‐systolic diameter (LVIDs), left ventricular end‐diastolic diameter (LVIDd), left ventricular end‐diastolic volume (LVEDV), and left ventricular end‐systolic volume (LVESV) were obtained. Left ventricular ejection fraction (EF) and fractional shortening (FS) were calculated using the formulas [(LVEDV – LVESV)/LVEDV × 100%] and [(LVIDd – LVIDs)/LVIDd × 100%], respectively.

### RNA Sequencing Assay

Oligo(dT) attached magnetic beads were used to purified mRNA from heart tissues in three replicates by eliminating rRNA and tRNA in a total amount of 2 µg RNA per sample. Purified mRNA was fragmented into small pieces with fragment buffer. Then, First‐strand cDNA was generated by random hexamer‐primed reverse transcription, followed by a second‐strand cDNA synthesis. Substantially, RNA index adapters and A‐tailing mix were incubated with cDNA, and then the fragments of cDNA were amplified by PCR. The PCR products were purified by AMPure XP Beads (Beckman). The double‐stranded PCR products were denatured by heating and circularized by the splint oligo sequence to generate the library. The final libraries were further amplified with phi29 to construct DNA nanoball (DNB). DNBs were loaded into the patterned nano‐array and paired‐end of 150 bp base reads were generated on T7 platform by Wuhan Benagen Technology Co., Ltd.

### RNA Sequencing Data Process and Analysis

The raw reads were filtered using trim‐galore, and the clean reads were aligned to mm10 by Hisat2 (v2.1.0). The genes with a fold change of > 1.5 and *p*‐value of < 0.05 were considered differentially expressed using the DESeq2 package. A Gene Ontology (GO) analysis was conducted to identify overrepresented biological processes.

### Cell Culture and Treatment

The primary human umbilical vein endothelial cells (HUVECs) were purchased from Procell Life Science Technology (Cat NO: CL‐0675) and nurtured in endothelial cell medium (Sciencell, 1001). SCH772984 (200 nm, MedChemExpress, USA) was administrated in HUVECs for 24 h. Saline solution was the vehicle as control. Using Rfect V2 (11 042, BAIDAI Biotech), control and Flt1 siRNA (Santa Cruz) were introduced into HUVECs. Total RNA or cell lysates were collected 48 h after siRNA transfection.

### TUNEL Staining

Cells were fixed with 4% paraformaldehyde at room temperature and then incubated with 0.2% Triton X‐100 at room temperature for 5 min for permeabilization. The cells were blocked with 5% BSA at room temperature for 1 hour. Substantially, cells were equilibrated and stained with terminal deoxynucleotidyl transferase (TdT) working solution and incubation without light at 37 °C for 1 h. Images were acquired using a laser scanning confocal microscope (LSM 800, Carl Zeiss, Germany).

### Cellular Thermal Shift Assay

After trypsin digestion, the collected cell suspension was transferred to a 15 mL centrifuge tube. The tube was centrifuged to remove the supernatant medium, and 1 mL of PBS with protease inhibitors was added to resuspend the cells. The EP tube was then heated in a water bath at the specified temperature for 3 minutes. After heating, the tube was incubated at room temperature for 5 min, followed by rapid freezing in liquid nitrogen. The cells were quickly frozen and thawed three times using liquid nitrogen and a constant‐temperature water bath, followed by a brief vortex. The tube was centrifuged, and the supernatant was discarded. RIPA cell lysis buffer containing protease inhibitors was added, followed by sonication and centrifugation for 15 min. Protein concentration was determined using the BCA method for subsequent experiments.

### Western Blot

To obtain the total protein, tissue or cultured cells were lysed with RIPA lysis (Beyotime, China) containing protease inhibitor cocktail (Beyotime, China). Protein samples were centrifugated (20 min, 13 000 × *g*) at 4 °C. The samples were quantified by BCA Protein Assay Kit (Beyotime, China). Then, protein samples (60 µg each) were separated by sodium dodecyl sulfate‐polyacrylamide gel electrophoresis (SDS‐PAGE) (10–12%) and transferred to PVDF membranes. After blocking, the membranes were incubated with primary antibodies. After washing with TBST (0.1% Tween in Tris), the membranes were incubated with the secondary antibodies against rabbit or mouse (1:8000, Proteintech, USA), and then detected by chemiluminescence using ECL luminescence solution (Tanon, China). The antibodies we used were p‐ERK1/2 (1:1000, AF1015, Affinity, USA), ERK1/2 (1:1000, AF0155, Affinity, USA), HIF1‐a (1:1000, BF8002, Affinity, USA), VE‐Cadherin (1:1000, AF6265, Affinity, USA), Bcl2 (1:1000, A19693, Abclonal, USA), Bax (1:5000, 60267‐1‐Ig, Proteintech, USA), Ubiquitin (1:2000, 10201‐2‐AP, Proteintech, USA), Flt1(1:1000, 13687‐1‐AP, Proteintech, USA) and β‐actin (1:20 000, 66240‐1‐Ig, Proteintech, USA), p‐P38(1:1000, AF4001, Affinity, USA), P38(1:2000, 14064‐1‐AP,Proteintech, USA), p‐JNK(1:2000, 80024‐1‐RR, Proteintech, USA), JNK(1:2000, 24164‐1‐AP, Proteintech, USA).

### Real‐Time Quantitative PCR

Total RNA samples of tissues and cells were extracted by TRIzol reagent (Vazyme, China). RNA samples were reverse transcribed using the HiScript Q RT SuperMix for qPCR (Vazyme, China). Real‐time quantitative PCR (qRT‐PCR) was performed by Hieff qPCR SYBR Green Master Mix (Yeasen, China). The relative RNA level was analyzed by using 2‐ΔΔct method, and β‐actin was used as an internal control. The primer pairs were synthesized by Tsingke Biotech Co., Ltd.

### Immunostaining

Cells were fixed with 4% PFA for 20 mins at room temperature. Then, 0.3% Triton X‐100 was added and allowed to stand for 1 h at room temperature. After 1 h of blocking with 10% BSA at room temperature, cells were incubated with primary antibodies overnight at 4 °C. After washing with PBS, cells were incubated with the secondary antibody at 37 °C for 1 h without light. After that. nuclei were labeled with DAPI (Beyotime, China) for 15 mins at room temperature without light. Photos were taken using a laser scanning confocal microscope (Handbuch LSM 800, Carl Zeiss, Germany). The antibodies used for immunostaining assay were: CD31 (1:800, ab94580, Abcam, England), SM22 (1:800, ab8295, Abcam, England), cTnT (1:800, ab8295, Abcam, England), SMA (1:200, 14395‐1‐AP, Proteintech, USA), IRG1 (1:200, ab222411, Abcam, England), CoraLite 488 (anti‐rabbit) (1:200, SA00013‐2, Proteintech, USA), CoraLite 594 (anti‐mouse) (1:500, SA00013‐3, Proteintech, USA).

### Scratch Assay

The scratch assay was performed as previously described. HUVECs were seeded at a density of 8 × 10^3^ cells cm^−2^ and incubated further until the cells on the surface reached ≈85% confluency. A scratch was made with a sterile 200 µL pipette tip. The cultures were then incubated for 16 h and analyzed by microscopy. Quantitative evaluation of the cell coverage was performed using the Image‐Pro Plus software (Media Cybernetics, Rockville, MD, USA).

### Transwell Assay

The migration of HUVECs was assessed using transwell with 8 µm pores (Corning, Corning, NY, USA). Briefly, serum‐starved HUVECs were seeded at 5 × 10^4^ cells in 100 µL in DMEM supplemented with 2% FBS and 1% penicillin‐streptomycin (P/S) onto the upper chamber. EVs and vehicle control (PBS) were added to the upper chambers. Cells were incubated for 18 h at 37 °C, and non‐migrated cells were removed from the inserts using cotton swabs. Migrated cells at the bottom of the membrane were fixed with 4% paraformaldehyde for 10 min. Cells were subsequently stained with 0.05% crystal violet staining solution for 15 min. After the inserts were completely dried, stained cells were visualized with a light microscope, and the stained areas were quantified using ImageJ software.

### Tube Formation Assay

The assay was performed as described previously. The plates were coated with Matrigel Membrane Matrix (Fisher Scientific, USA). The coated plates were overlayed with HUVEC cells (density of 5 × 10^3^ cells cm^−2^). After 12 h of incubation in Endothelial Cell Basal Medium, cells were stained with calcein‐AM and processed through critical point fixation steps.

### Co‐Immunoprecipitation

To determine the interaction between proteins, PierceTM CO‐Immunoprecipitation Kit (Thermo Fisher, America) was used. Cultured cells were lysed in lysis buffer containing 1% protease inhibitor (Roche, Switzerland) for 20 min in an ice bath. Protein samples were obtained by centrifugation (15 min, 13 000 × *g*) at 4 °C. After incubating with control agarose resin for 1 h at a 4 °C table concentrator, the final protein samples were obtained by centrifugation (1 min, 1000 × *g*) at 4 °C. The antibodies (10 µg) were pretreated by incubating with AminoLink Plus coupling resin for 1 h at room temperature and then added to protein samples and incubated overnight at 4 °C. After three times washing, co‐immunoprecipitation products were obtained with elution buffer. The co‐immunoprecipitation products were analyzed by Western blot. The antibodies used for co‐immunoprecipitation were Ubiquitin (10201‐2‐AP, Proteintech, USA) and Flt1(13687‐1‐AP, Proteintech, USA).

### Statistical Analysis

All statistical calculations were performed using Prism software (version 8.3.0, GraphPad, America). Data are expressed as mean ± SD. In data statistics, two‐tailed Student's t‐test was used to compare two groups. One‐way analysis of variance (ANOVA) followed by Tukey's post‐hoc multi‐comparison test was used to compare differences among multiple groups. Statistical analyses comparing with more than two groups were done using a two‐way analysis of variance (ANOVA) followed by Bonferroni's post hoc test. In all cases, significance was defined as *p* ≤ 0.05.

## Conflict of Interest

The authors declare no conflict of interest.

## Supporting information



Supporting Information

## Data Availability

The data that support the findings of this study are available from the corresponding author upon reasonable request.

## References

[1] J. L. Zweier , J. Biol. Chem. 1988, 263, 1353. 2826476

[2] D. J. Hausenloy , D. M. Yellon , J. Mol. Cell. Cardiol. 2003, 35, 339. 12689812 10.1016/s0022-2828(03)00043-9

[3] H. M. Piper , D. Garcia‐Dorado , M. Ovize , Cardiovasc. Res. 1998, 38, 291. 9709390 10.1016/s0008-6363(98)00033-9

[4] J. J. Lemasters , J. M. Bond , E. Chacon , I. S. Harper , S. H. Kaplan , H. Ohata , D. R. Trollinger , B. Herman , W. E. Cascio , EXS 1996, 76, 99. 8805791 10.1007/978-3-0348-8988-9_7

[5] L. Deveza , J. Choi , F. Yang , Theranostics 2012, 2, 801. 22916079 10.7150/thno.4419 PMC3425124

[6] L. Tan , L. Long , H. Li , W. Yang , Y. Peng , J. Lu , F. Liao , X. Ma , H. Qu , C. Fu , S. Zhang , Front. Cell Dev. Biol. 2022, 10, 1095623. 36568984 10.3389/fcell.2022.1095623 PMC9780500

[7] A. V. Ferreira , M. G. Netea , J. Dominguez‐Andres , Aging 2019, 11, 3898. 31235675 10.18632/aging.102057 PMC6629002

[8] A. Michelucci , T. Cordes , J. Ghelfi , A. Pailot , N. Reiling , O. Goldmann , T. Binz , A. Wegner , A. Tallam , A. Rausell , M. Buttini , C. L. Linster , E. Medina , R. Balling , K. Hiller , Proc. Natl. Acad. Sci. U. S. A. 2013, 110, 7820. 23610393 10.1073/pnas.1218599110 PMC3651434

[9] M. Sano , T. Tanaka , H. Ohara , Y. Aso , Appl. Microbiol. Biotechnol. 2020, 104, 9041. 32945901 10.1007/s00253-020-10908-1

[10] E. L. Mills , D. G. Ryan , H. A. Prag , D. Dikovskaya , D. Menon , Z. Zaslona , M. P. Jedrychowski , A. S. H. Costa , M. Higgins , E. Hams , J. Szpyt , M. C. Runtsch , M. S. King , J. F. McGouran , R. Fischer , B. M. Kessler , A. F. McGettrick , M. M. Hughes , R. G. Carroll , L. M. Booty , E. V. Knatko , P. J. Meakin , M. L. J. Ashford , L. K. Modis , G. Brunori , D. C. Sévin , P. G. Fallon , S. T. Caldwell , E. R. S. Kunji , E. T. Chouchani , et al., Nature 2018, 556, 113. 29590092 10.1038/nature25986 PMC6047741

[11] X. Zhu , Y. Guo , Z. Liu , J. Yang , H. Tang , Y. Wang , Sci. Rep. 2021, 11, 18173. 34518559 10.1038/s41598-021-97352-x PMC8438069

[12] C. Tang , X. Wang , Y. Xie , X. Cai , N. Yu , Y. Hu , Z. Zheng , Cell. Physiol. Biochem. 2018, 51, 979. 30466076 10.1159/000495400

[13] M. Bambouskova , L. Gorvel , V. Lampropoulou , A. Sergushichev , E. Loginicheva , K. Johnson , D. Korenfeld , M. E. Mathyer , H. Kim , L. Huang , D. Duncan , H. Bregman , A. Keskin , A. Santeford , R. S. Apte , R. Sehgal , B. Johnson , G. K. Amarasinghe , M. P. Soares , T. Satoh , S. Akira , T. Hai , C. de Guzman Strong , K. Auclair , T. P. Roddy , S. A. Biller , M. Jovanovic , E. Klechevsky , K. M. Stewart , G. J. Randolph , et al., Nature 2018, 556, 501. 29670287 10.1038/s41586-018-0052-z PMC6037913

[14] V. Lampropoulou , A. Sergushichev , M. Bambouskova , S. Nair , E. E. Vincent , E. Loginicheva , L. Cervantes‐Barragan , X. Ma , S. C. Huang , T. Griss , C. J. Weinheimer , S. Khader , G. J. Randolph , E. J. Pearce , R. G. Jones , A. Diwan , M. S. Diamond , M. N. Artyomov , Cell Metab. 2016, 24, 158. 27374498 10.1016/j.cmet.2016.06.004 PMC5108454

[15] T. Cordes , A. Lucas , A. S. Divakaruni , A. N. Murphy , P. Cabrales , C. M. Metallo , Mol. Metab. 2020, 32, 122. 32029222 10.1016/j.molmet.2019.11.019 PMC6961711

[16] K. A. Sun , Y. Li , A. Y. Meliton , P. S. Woods , L. M. Kimmig , R. Cetin‐Atalay , R. B. Hamanaka , G. M. Mutlu , Elife 2020, 9. 10.7554/eLife.54877 PMC7185992 32255424

[17] Q. Wang , X. L. Li , Y. Mei , J. C. Ye , W. Fan , G. H. Cheng , M. S. Zeng , G. K. Feng , J. Mol. Med. 2020, 98, 1457. 32840638 10.1007/s00109-020-01963-2

[18] I. C. Henderson , T. E. Frei 3rd, N. Engl. J. Med. 1979, 300, 310. 759884 10.1056/NEJM197902083000610

[19] Q. Shan , X. Li , M. Zheng , X. Lin , G. Lu , D. Su , X. Lu , Biochem. Biophys. Res. Commun. 2019, 517, 538. 31376936 10.1016/j.bbrc.2019.07.046

[20] V. C. Ganta , M. H. Choi , A. Kutateladze , T. E. Fox , C. R. Farber , B. H. Annex , Circulation 2017, 135, 2403. 28356443 10.1161/CIRCULATIONAHA.116.025490 PMC5503157

[21] E. T. Chouchani , V. R. Pell , E. Gaude , D. Aksentijevic , S. Y. Sundier , E. L. Robb , A. Logan , S. M. Nadtochiy , E. N. J. Ord , A. C. Smith , F. Eyassu , R. Shirley , C. Hu , A. J. Dare , A. M. James , S. Rogatti , R. C. Hartley , S. Eaton , A. S. H. Costa , P. S. Brookes , S. M. Davidson , M. R. Duchen , K. Saeb‐Parsy , M. J. Shattock , A. J. Robinson , L. M. Work , C. Frezza , T. Krieg , M. P. Murphy , Nature 2014, 515, 431. 25383517 10.1038/nature13909 PMC4255242

[22] J. L. Martin , A. S. H. Costa , A. V. Gruszczyk , T. E. Beach , F. M. Allen , H. A. Prag , E. C. Hinchy , K. Mahbubani , M. Hamed , L. Tronci , E. Nikitopoulou , A. M. James , T. Krieg , A. J. Robinson , M. M. Huang , S. T. Caldwell , A. Logan , L. Pala , R. C. Hartley , C. Frezza , K. Saeb‐Parsy , M. P. Murphy , Nat. Metab. 2019, 1, 966. 32395697 10.1038/s42255-019-0115-y PMC7212038

[23] Z. Hu , D. Xu , H. Meng , W. Liu , Q. Zheng , J. Wang , Biochem. Pharmacol. 2024, 220, 115992. 38128618 10.1016/j.bcp.2023.115992

[24] D. Xu , B. Shan , B. Lee , K. Zhu , T. Zhang , H. Sun , M. Liu , L. Shi , W. Liang , L. Qian , J. Xiao , L. Wang , L. Pan , D. Finley , J. Yuan , Elife 2015, 4, e10510. 26523394 10.7554/eLife.10510 PMC4733041

[25] Q. Li , Y. Li , B. Gu , L. Fang , P. Zhou , S. Bao , L. Huang , X. Dai , J. Biol. Chem. 2015, 290, 21553. 26170450 10.1074/jbc.M115.639419 PMC4571880

[26] J. Hu , Q. Yan , C. Shi , Y. Tian , P. Cao , W. Yuan , Am. J. Transl. Res. 2017, 9, 79. 28123635 PMC5250705

[27] F. J. Wu , Y. Xue , X. F. Liu , C. H. Xue , J. F. Wang , L. Du , K. Takahashi , Y. M. Wang , Neurochem. Int. 2014, 64, 9. 24231470 10.1016/j.neuint.2013.10.015

[28] A. S. Vrablic , C. D. Albright , C. N. Craciunescu , R. I. Salganik , S. H. Zeisel , FASEB J. 2001, 15, 1739. 11481221 10.1096/fj.00-0300com

[29] Y. Ding , P. Wang , C. Li , Y. Zhang , C. Yang , X. Zhou , X. Wang , Z. Su , W. Ming , L. Zeng , Y. Shi , C. Li , X. Kang , Int. J. Mol. Sci. 2023, 24, 13474. 37686278 10.3390/ijms241713474 PMC10487490

[30] R. Valenzuela , L. A. Videla , Nutrients 2020, 12, 499. 32075238 10.3390/nu12020499 PMC7071322

[31] R. Valenzuela , P. Illesca , F. Echeverria , A. Espinosa , M. A. Rincon‐Cervera , M. Ortiz , M. C. Hernandez‐Rodas , A. Valenzuela , L. A. Videla , Food Funct. 2017, 8, 1526. 28386616 10.1039/c7fo00090a

[32] M. Ortiz , S. A. Soto‐Alarcon , P. Orellana , A. Espinosa , C. Campos , S. Lopez‐Arana , M. A. Rincon , P. Illesca , R. Valenzuela , L. A. Videla , Dig. Liver Dis. 2020, 52, 895. 32620521 10.1016/j.dld.2020.04.019

[33] Z. Yi , M. Deng , M. J. Scott , G. Fu , P. A. Loughran , Z. Lei , S. Li , P. Sun , C. Yang , W. Li , H. Xu , F. Huang , T. R. Billiar , Hepatology 2020, 72, 1394. 31997373 10.1002/hep.31147 PMC7702080

[34] H. Liu , Y. Feng , M. Xu , J. Yang , Z. Wang , G. Di , Cell Commun. Signal 2018, 16, 81. 30442144 10.1186/s12964-018-0294-2 PMC6238317

[35] R. He , B. Liu , R. Xiong , B. Geng , H. Meng , W. Lin , B. Hao , L. Zhang , W. Wang , W. Jiang , N. Li , Q. Geng , Cell Death Discov. 2022, 8, 43. 35110526 10.1038/s41420-021-00807-3 PMC8810876

[36] H. A. Prag , D. Aksentijevic , A. Dannhorn , A. V. Giles , J. F. Mulvey , O. Sauchanka , L. Du , G. Bates , J. Reinhold , D. Kula‐Alwar , Z. Xu , L. Pellerin , R. J. A. Goodwin , M. P. Murphy , T. Krieg , Circ. Res. 2022, 131, 528. 35959683 10.1161/CIRCRESAHA.121.320717 PMC9426742

[37] A. N. Booth , J. Taylor , R. H. Wilson , F. Deeds , J. Biol. Chem. 1952, 195, 697. 14946179

[38] W. Zhang , H. T. Liu , Cell Res. 2002, 12, 9. 11942415 10.1038/sj.cr.7290105

[39] M. Shibuya , Genes Cancer 2011, 2, 1097. 22866201 10.1177/1947601911423031 PMC3411125

[40] A. K. Olsson , A. Dimberg , J. Kreuger , L. Claesson‐Welsh , Nat. Rev. Mol. Cell Biol. 2006, 7, 359. 16633338 10.1038/nrm1911

[41] S. Koch , S. Tugues , X. Li , L. Gualandi , L. Claesson‐Welsh , Biochem. J. 2011, 437, 169. 21711246 10.1042/BJ20110301

[42] L. Ye , S. He , X. Mao , Y. Zhang , Y. Cai , S. Li , Front. Immunol. 2020, 11, 1193. 32676077 10.3389/fimmu.2020.01193 PMC7333353

[43] H. Wang , Z. Xi , L. Deng , Y. Pan , K. He , Q. Xia , Int. J. Med. Sci. 2021, 18, 1104. 33526969 10.7150/ijms.52691 PMC7847630

[44] S. Shen , J. Li , Z. Wei , Y. Liu , L. Kang , R. Gu , X. Sun , B. Xu , Q. Li , Biol. Direct 2024, 19, 86. 39350193 10.1186/s13062-024-00521-x PMC11441264

[45] E. Zajac , B. Schweighofer , T. A. Kupriyanova , A. Juncker‐Jensen , P. Minder , J. P. Quigley , E. I. Deryugina , Blood 2013, 122, 4054. 24174628 10.1182/blood-2013-05-501494 PMC3862278

[46] S. Liu , J. Chen , J. Shi , W. Zhou , L. Wang , W. Fang , Y. Zhong , X. Chen , Y. Chen , A. Sabri , S. Liu , Basic Res. Cardiol. 2020, 115, 22. 32112145 10.1007/s00395-020-0781-7

[47] S. Gong , M. Zhai , J. Shi , G. Yu , Z. Lei , Y. Shi , Y. Zeng , P. Ju , N. Yang , Z. Zhang , D. Zhang , J. Zhuang , Q. Yu , X. Zhang , W. Jian , W. Wang , W. Peng , Cell Death Differ. 2024, 31, 239. 38182899 10.1038/s41418-023-01252-8 PMC10850484

[48] E. Patino‐Martinez , S. Nakabo , K. Jiang , C. Carmona‐Rivera , W. L. Tsai , D. Claybaugh , Z. X. Yu , A. Romero , E. Bohrnsen , B. Schwarz , M. A. Solis‐Barbosa , L. P. Blanco , M. Naqi , Y. Temesgen‐Oyelakin , M. Davis , Z. Manna , S. Gupta , N. Mehta , F. Naz , S. dell'Orso , S. Hasni , M. J. Kaplan , J Immunol. 2024, 213, 419. 38949522 10.4049/jimmunol.2400241 PMC11817569

[49] X. Duan , M. Hu , L. Yang , S. Zhang , B. Wang , T. Li , Y. Tan , Y. Li , X. Liu , Z. Zhan , Biochem. Pharmacol. 2023, 213, 115614. 37209857 10.1016/j.bcp.2023.115614

[50] Q. Luo , W. Sun , Z. Li , J. Sun , Y. Xiao , J. Zhang , C. Zhu , B. Liu , J. Ding , Biomaterials 2023, 303, 122368. 37977009 10.1016/j.biomaterials.2023.122368

[51] Q. Luo , Z. Li , B. Liu , J. Ding , Expert. Opin. Drug Deliv 2024, 21, 1463. 39323051 10.1080/17425247.2024.2409906

