# Supplementary material for: 4‐Octyl Itaconate Alleviates Myocardial Ischemia‐Reperfusion Injury Through Promoting Angiogenesis via ERK Signaling Activation
Source: Adv Sci (Weinh). 2025 Jan 21;12(10):2411554. doi: 10.1002/advs.202411554 (PMC11904966; doi:10.1002/advs.202411554)

**Supplementary Information**

**SUPPLEMENTARY TABLE**

**Table S1. Sequences of mouse oligonucleotide primers used for real-time quantitative PCR**

| Anp | Mus | Forward | 5’-CGTCTTGGCCTTTTGGCTTC-3’ |
| --- | --- | --- | --- |
|  |  | Reverse | 5’-GGTGGTCTAGCAGGTTCTTGAAA-3’ |
| Bnp | Mus | Forward | 5’-CTCTCCCCCGCAAAAGAAAAA-3’ |
|  |  | Reverse | 5’-CGGAACATCTCGAAGCGTTTA-3’ |
| Bax | Mus | Forward | 5’-CCAAGAAGCTGAGCGAGTGT-3’ |
|  |  | Reverse | 5’-CACGTCAGCAATCATCCTCTG-3’ |
| Bcl2 | Mus | Forward | 5’-TGGCATCTTCTCCTTCCAGC-3’ |
|  |  | Reverse | 5’-ACGTCCTGGCAGCCATGTC-3’ |
| Cdh5 | Mus | Forward | 5’-CCTGAGGCAATCAACTGTGC-3’ |
|  |  | Reverse | 5’-GGAGGAGCTGATCTTGTCCG -3’ |
| eNOS | Mus | Forward | 5’-CCTGAGCAGCACAAGAGCTA-3’ |
|  |  | Reverse | 5’-TCGAGCAAAGGCACAGAAGT-3’ |
| Flt1 | Mus | Forward | 5’-CACCCCTGTCACCACAATCA-3’ |
|  |  | Reverse | 5’-CACCAATGTGCTAACCGTCTTA-3’ |
| FLT1 | Homo | Forward | 5’-TTTAGGACCAGGAAGCAGCAC-3’ |
|  |  | Reverse | 5’-GACTTGTCCGAGGTTCCTTGA-3’ |
| Irg1 | Mus | Forward | 5’-AGTTTTCTGGCCTCGACCTG-3’ |
|  |  | Reverse | 5’-AGAGGGAGGGTGGAATCTCT-3’ |
| Mmp2 | Mus | Forward | 5’-CAAGTTCCCCGGCGATGTC-3’ |
|  |  | Reverse | 5’-TTCTGGTCAAGGTCACCTGTC-3’ |
| Mmp9 | Mus | Forward | 5’-CTGGACAGCCAGACACTAAAG-3’ |
|  |  | Reverse | 5’-CTCGCGGCAAGTCTTCAGAG-3’ |
| Vegfa | Mus | Forward | 5’-ACTGGACCCTGGCTTTACTG-3’ |
|  |  | Reverse | 5’-TCTGCTCTCCTTCTGTCGTG-3’ |
| Vegfr2 | Mus | Forward | 5’-CTGTGAACGCTTGCCTTAT-3’ |
|  |  | Reverse | 5’-CAACATCTTGACGGCTACTG-3’ |
| β-Mhc | Mus | Forward | 5’- TCCTCTAGAACAGCAGCGGG-3’ |
|  |  | Reverse | 5’-TCTCGGAGCCACCTTGGAA-3’ |
| β-actin | Mus | Forward | 5’-GGCTGTATTCCCCTCCATCG-3’ |
|  |  | Reverse | 5’-CCAGTTGGTAACAATGCCATGT-3’ |
| β-actin | Homo | Forward | 5’-CGCGAGAAGATGACCCAGAT-3’ |
|  |  | Reverse | 5’-TCACCGGAGTCCATCACGAT-3’ |

**Table S2. 4-OI improves cardiac function in IR male mice.**

| **Group** | **Sham+Veh**  **(n=6)** | **Sham+4OI**  **(n=6)** | **IR+Veh**  **(n=6)** | **IR+ 4OI**  **(n=6)** |
| --- | --- | --- | --- | --- |
| EF, % | 62.08±2.30 | 64.75±3.77 | 50.49±3.94^*^ | 60.79±3.35^#^ |
| FS, % | 32.74±1.64 | 34.43±2.86 | 24.96±2.14^*^ | 31.61±2.41^#^ |
| IVS;d,mm | 0.70±0.06 | 0.71±0.04 | 0.69±0.10 | 0.69±0.05 |
| IVS;s,mm | 1.08±0.08 | 1.11±0.05 | 0.93±0.15 | 1.03±0.09 |
| LVIDd, mm | 3.62±0.20 | 3.25±0.38 | 3.39±0.44 | 3.28±0.23 |
| LVIDs, mm | 2.43±0.15 | 2.13±0.24 | 2.55±0.40 | 2.24±0.11 |
| LVPW;d,mm  LVPW;s,mm | 0.74±0.13  1.16±0.11 | 0.88±0.13  1.22±0.08 | 0.69±0.10  0.95±0.13^*^ | 0.77±0.14  1.15±0.15 |

^*^*p* < 0.05 versus Sham+Veh group; ^#^*p* < 0.05 versus IR+4OI group.

They were analyzed by using two-way ANOVA, followed by Bonferroni’s post hoc analysis. The data are expressed as means ± SD.

**Table S3. Acod1-deficiency aggravates cardiac function in IR mice.**

| **Group** | **Sham+WT**  **(n=6)** | **Sham+KO**  **(n=6)** | **IR+WT**  **(n=6)** | **IR+KO**  **(n=6)** |  |
| --- | --- | --- | --- | --- | --- |
| EF, % | | 61.99±5.44 | 61.42±4.51 | 52.84±2.45^*^ | 43.26±1.47^#^ |
| FS, % | | 32.77±3.97 | 32.20±3.02 | 26.41±1.57^*^ | 20.95±0.93^#^ |
| IVS;d,mm | | 0.67±0.13 | 0.67±0.05 | 0.69±0.07 | 0.74±0.05 |
| IVS;s,mm | | 1.06±0.16 | 1.07±0.10 | 1.03±0.07 | 1.00±0.11 |
| LVIDd, mm | | 3.58±0.38 | 3.45±0.42 | 3.41±0.49 | 3.87±0.37 |
| LVIDs, mm | | 2.42±0.35 | 2.35±0.34 | 2.51±0.35 | 3.05±0.28^#^ |
| LVPW;d,mm  LVPW;s,mm | | 0.76±0.16  1.15±0.11 | 0.74±0.06  1.07±0.12 | 0.78±0.07  1.06±0.06 | 0.78±0.09  1.01±0.06 |

^*^*p* < 0.05 versus Sham+WT group; ^#^*p* < 0.05 versus IR+WT group.

They were analyzed by using two-way ANOVA, followed by Bonferroni’s post hoc analysis. The data are expressed as means ± SD.

**Table S4. 4-OI alleviates cardiac damage in *Irg1*-deficient mice following IR injury**

| **Group** | **KOSham+Veh**  **(n=6)** | **KOSham+4-OI**  **(n=6)** | **KOIR+Veh**  **(n=6)** | **KOIR+4-OI**  **(n=6)** |  |
| --- | --- | --- | --- | --- | --- |
| EF, % | | 59.19±3.37 | 62.65±4.30 | 42.20±2.21^*^ | 51.75±4.31^#^ |
| FS, % | | 30.93±2.08 | 33.15±3.03 | 20.26±1.17^*^ | 25.82±2.58^#^ |
| IVS;d,mm | | 0.63±0.03 | 0.77±0.03 | 0.78±0.07^*^ | 0.71±0.07 |
| IVS;s,mm | | 1.00±0.05 | 1.15±0.05 | 1.04±0.13 | 1.01±0.13 |
| LVIDd, mm | | 3.93±0.45 | 3.55±0.42 | 3.68±0.28 | 3.47±0.32 |
| LVIDs, mm | | 2.72±0.38 | 2.38±0.34 | 2.94±0.24 | 2.58±0.30^#^ |
| LVPW;d,mm  LVPW;s,mm | | 0.73±0.06  1.06±0.08 | 0.78±0.08  1.14±0.05 | 0.83±0.10  1.07±0.12 | 0.84±0.10  1.10±0.17 |

^*^*p* < 0.05 versus KOSham+Veh group; ^#^*p* < 0.05 versus KOIR+Veh group.

They were analyzed by using two-way ANOVA, followed by Bonferroni’s post hoc analysis. The data are expressed as means ± SD.

**Table S5. 4-OI improves cardiac function in IR female mice.**

| **Group** | **Sham+Veh**  **(n=6)** | **Sham+4OI**  **(n=6)** | **IR+Veh**  **(n=6)** | **IR+ 4OI**  **(n=6)** |
| --- | --- | --- | --- | --- |
| EF, % | 61.62±2.45 | 64.17±1.08 | 47.38±3.50^*^ | 58.81±2.26^#^ |
| FS, % | 32.60±1.58 | 34.18±0.84 | 23.09±2.07^*^ | 30.69±1.48^#^ |
| IVS;d,mm | 0.71±0.05 | 0.72±0.07 | 0.75±0.14 | 0.79±0.06 |
| IVS;s,mm | 1.09±0.06 | 1.06±0.12 | 1.05±0.21 | 1.17±0.10 |
| LVIDd, mm | 3.90±0.38 | 3.55±0.28 | 3.35±0.30^*^ | 3.97±0.23^#^ |
| LVIDs, mm | 2.63±0.30 | 2.34±0.18 | 2.58±0.24 | 2.75±0.19 |
| LVPW;d,mm  LVPW;s,mm | 0.71±0.04  1.12±0.05 | 0.72±0.06  1.09±0.08 | 0.77±0.14  0.99±0.16 | 0.79±0.07  1.17±0.09 |

^*^*p* < 0.05 versus Sham+Veh group; ^#^*p* < 0.05 versus IR+4OI group.

They were analyzed by using two-way ANOVA, followed by Bonferroni’s post hoc analysis. The data are expressed as means ± SD.

**SUPPLEMENTARY FIGURE**

**Supplementary Figure 1**

**
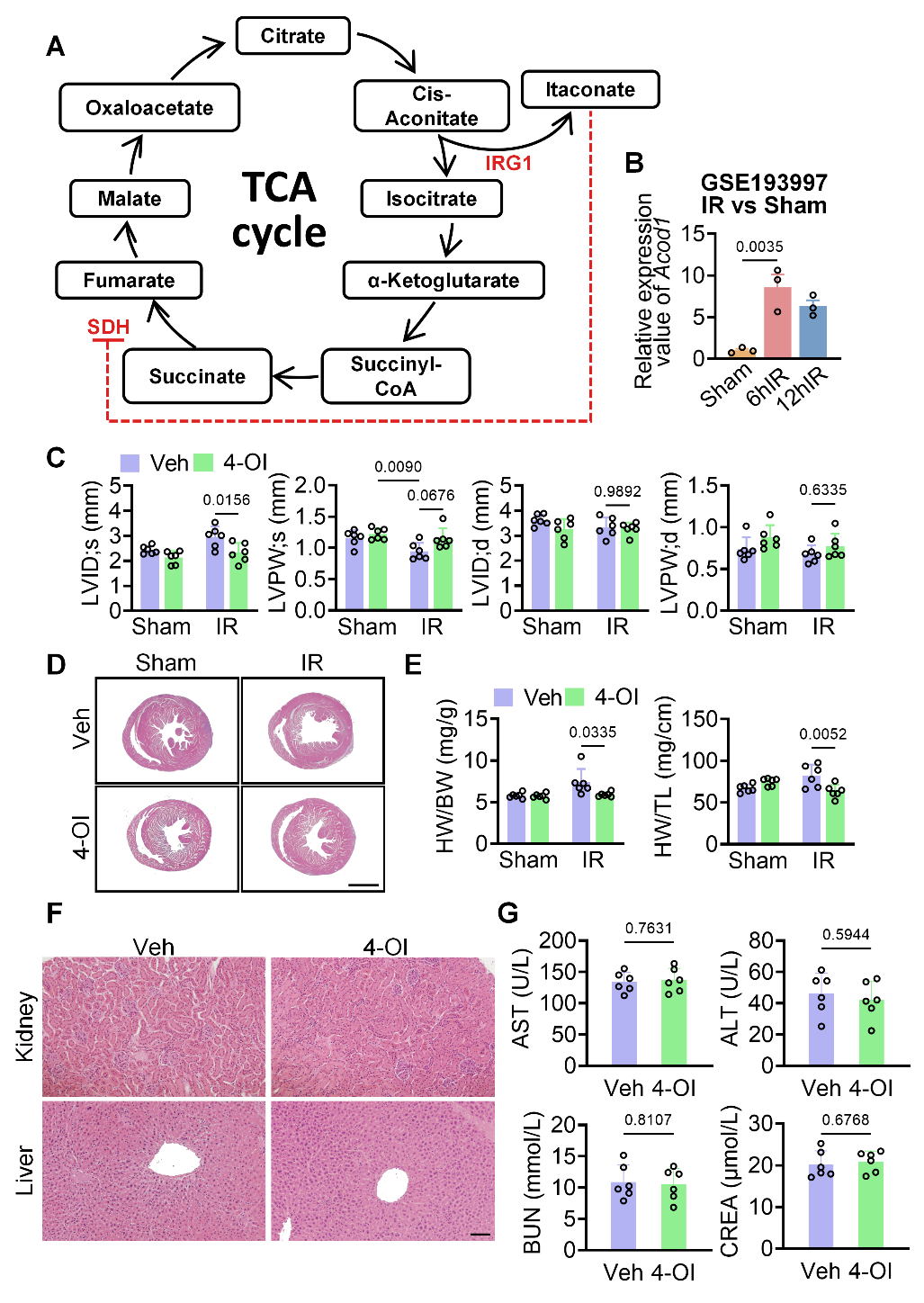
**

**Figure S1. 4-OI reverses changed cardiac function after myocardial ischemia-reperfusion injury.A**, The diagram of the TCA cycle. **B**, The relative expression value of Acod1 in GSE193997. **C**, 8-week-old C57BL/6 (WT) mice were subjected to IR injury and treated with 4-OI, then echocardiography was performed (n=6). **D**, Representative hematoxylin-eosin (H&E) staining from heart tissues (n=3). Scale bar = 2mm. **E**, The ratios for heart weight/body weight (HW/BW, mg/g) and heart weight/tibia length (HW/TL, mg/cm) post sham or IR surgery were shown (n=6). **F**, HE staining of mouse liver and kidney after solvent or 4-OI administration (n=3). Scale bar = 100μm. **G**, The concentration changes of ALT, AST, BUN, and CREA in serum of mice treated with solvent or 4-OI (n=6). All data are presented as mean ± SD, and P-values are calculated using one-way ANOVA with Bonferroni correction.

**Supplementary Figure 2**

**
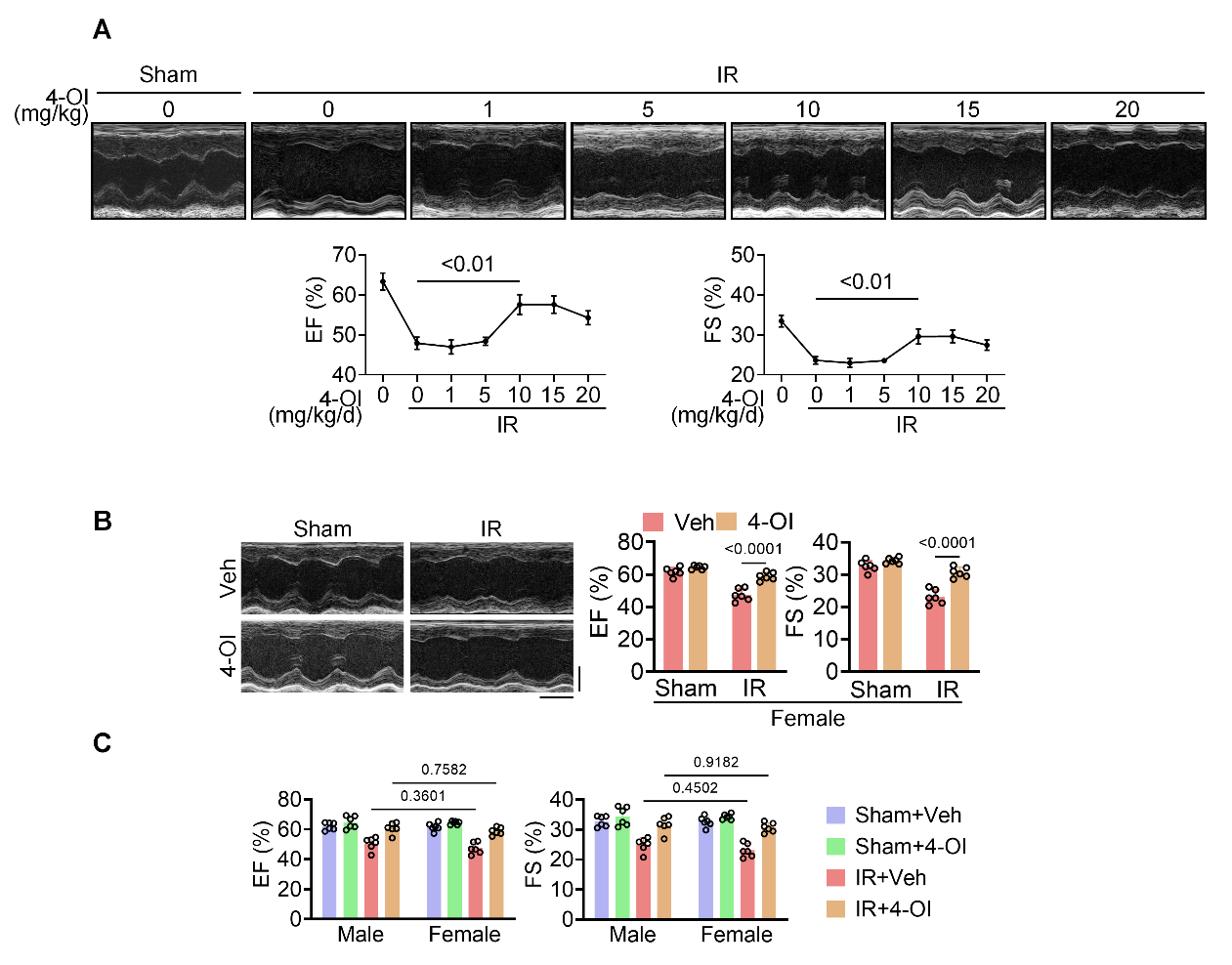
**

**Figure S2. 4-OI reverses changed cardiac function after myocardial ischemia-reperfusion injury.A**, 8-week-old C57BL/6 (WT) male mice were subjected to IR injury and treated with or without different doses of 4-OI. Then echocardiography was performed (n=3). **B**, 8-week-old C57BL/6 (WT) female mice were subjected to IR injury and treated with 4-OI, then echocardiography was performed (n=6). All data are presented as mean ± SD, and P-values are calculated using one-way ANOVA with Bonferroni correction.

**Supplementary Figure 3**

**
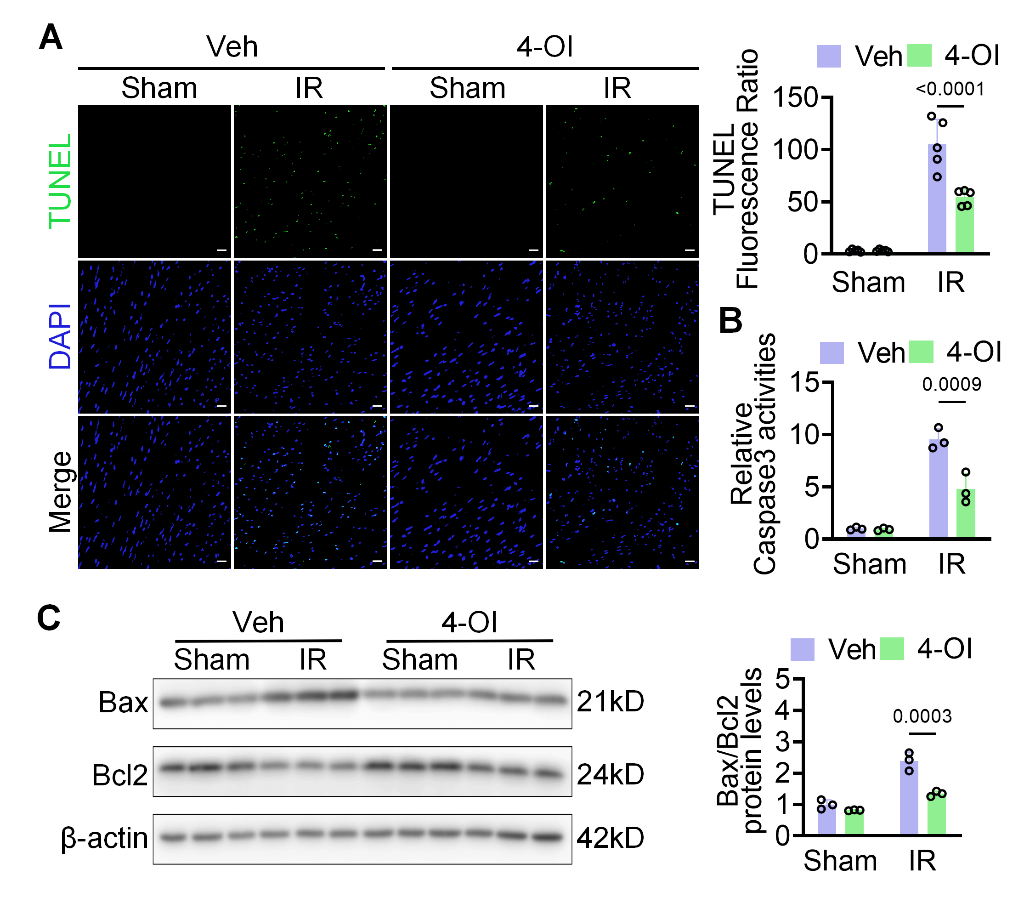
**

**Figure S3. The apoptosis level of cardiac tissue changed after 4-OI administration during IR injury.A**, Representative images of TUNEL (green) staining in heart tissue (n=5). Scale bar = 20 μm. **B**, The relative Caspase 3 activities in hearts from different groups were detected (n=3). **C**, The expression of Bax and Bcl2 at protein levels was detected in heart tissues (n=3). All data are presented as mean ± SD, and P-values are calculated using one-way ANOVA with Bonferroni correction.

**Supplementary Figure 4**

**
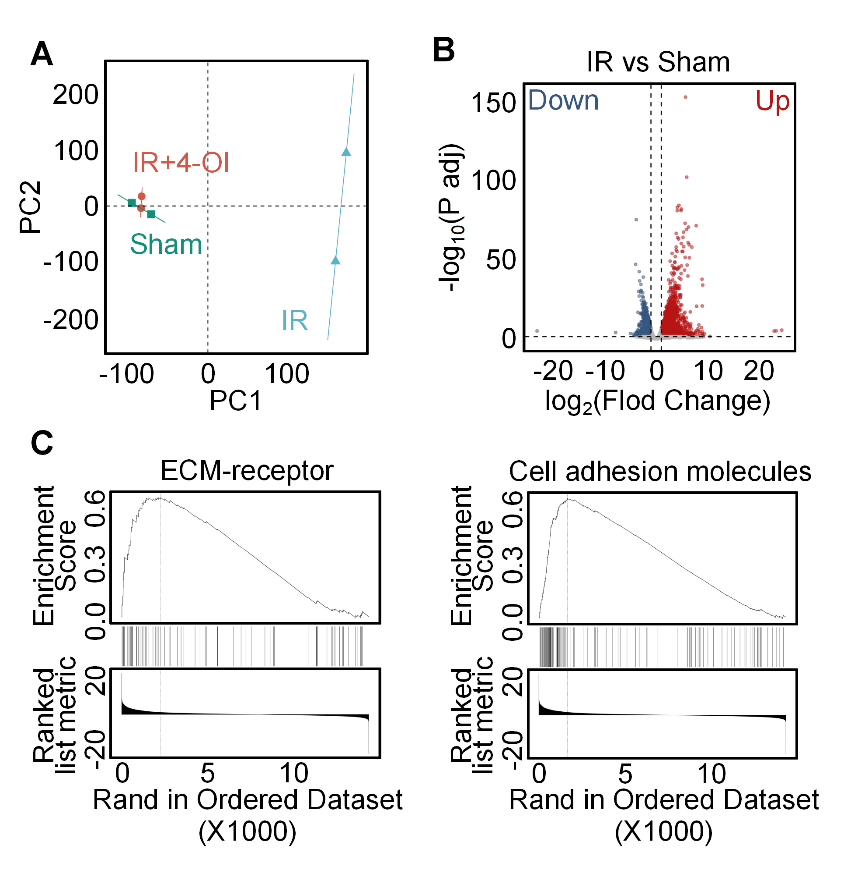
**

**Figure S4. Variation of heart tissues for RNAseq.A**, Principal component analysis (PCA) indicated the consistency of samples for each group. **B**, Volcano plot showing the difference of gene expression in hearts between WT mice subjected to IR operation and WT mice subjected to Sham operation. **C**, GSEA enrichment plot of ECM-receptor and Cell adhesion molecules in analysis between IR operation and Sham operation.

**Supplementary Figure 5**

**
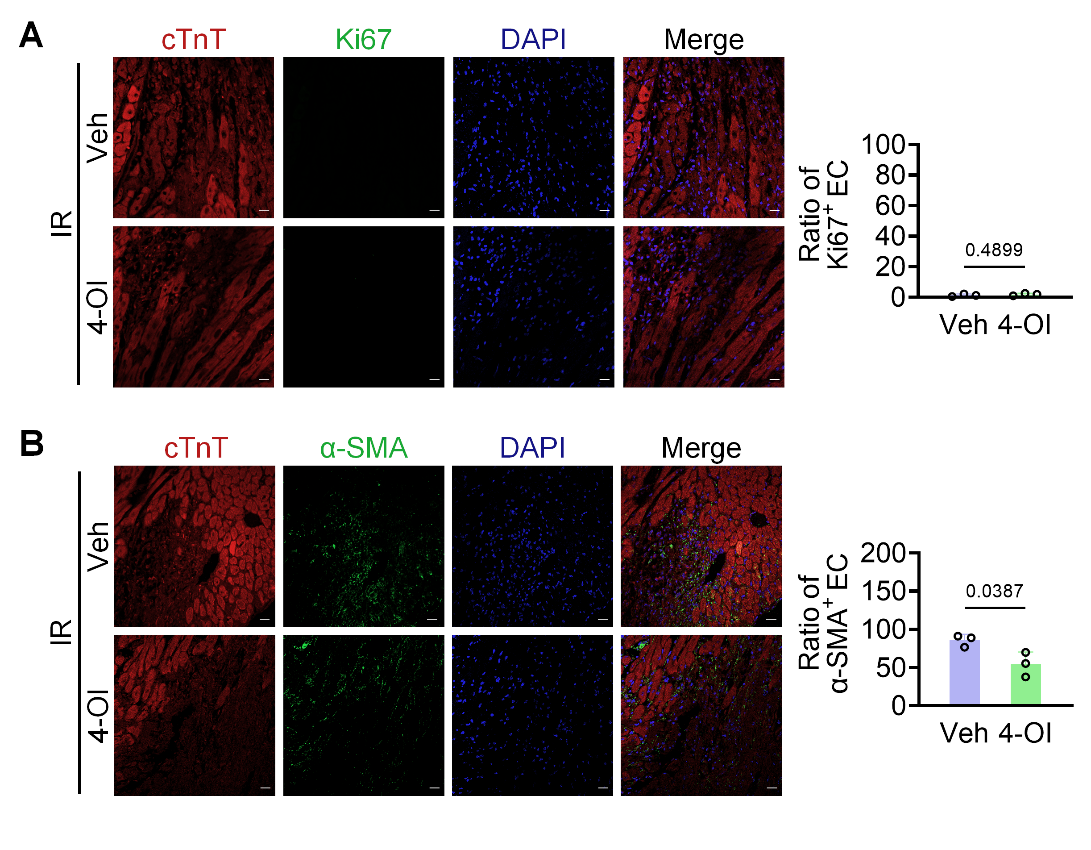
**

**Figure S5. The changes of cardiomyocytes and fibroblasts in cardiac tissue.** A, Immunofluorescent staining was performed with antibodies against Ki67 (green) and cardiomyocytic marker cTnT (red) (n=3). Scale bar = 20 μm. B, Immunofluorescent staining was performed with antibodies against α-SMA (green) and cardiomyocytic marker cTnT (red) (n=3). Scale bar = 20 μm. All data are presented as mean ± SD, and P-values are calculated using student t-test.

**Supplementary Figure 6**

**
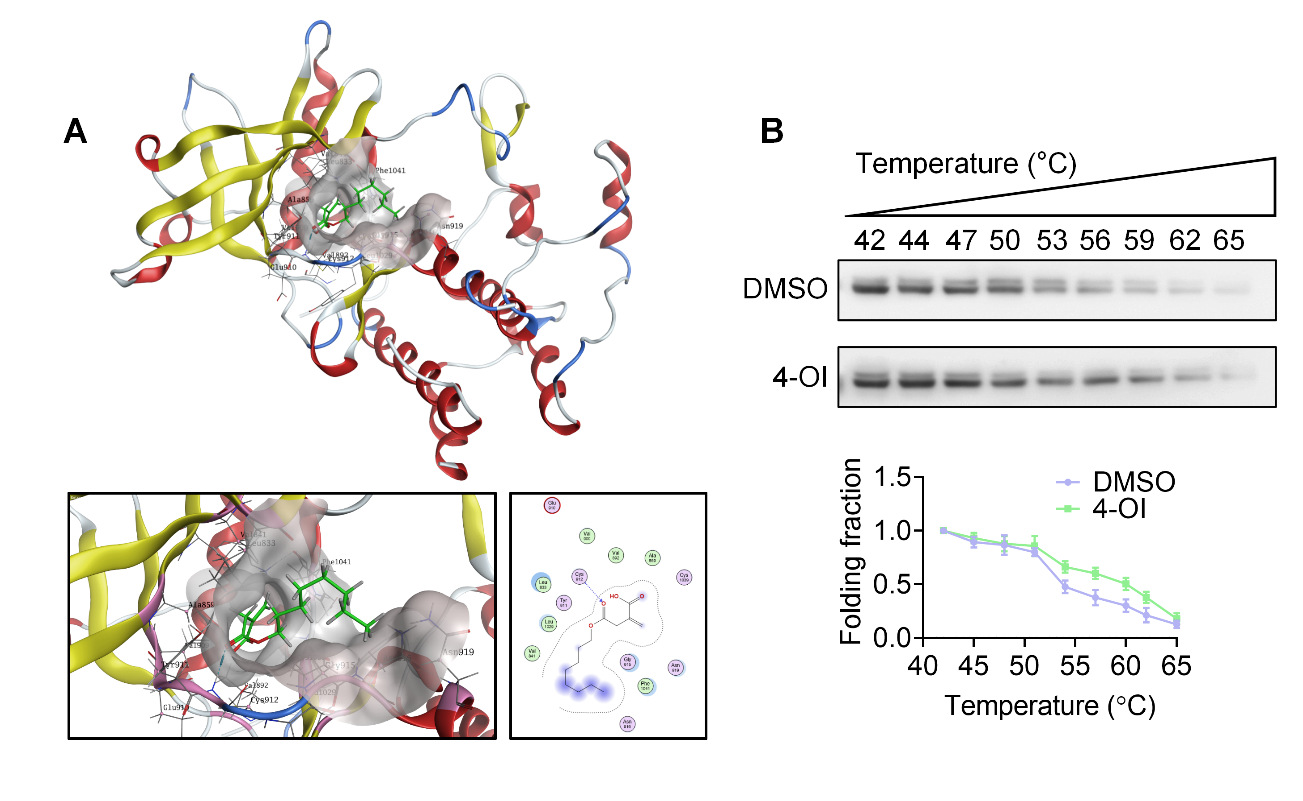
**

**Figure S6. The binding ability of 4-OI and Flt1.A**, The molecular docking plot of 4-OI and Flt1. **B**, Cellular Thermal Shift Assay (CETSA) represents a western blot strip plot and a folding line graph of Flt1 protein with temperature change. (n=3). All data are presented as mean ± SD.

**Supplementary Figure 7**

**
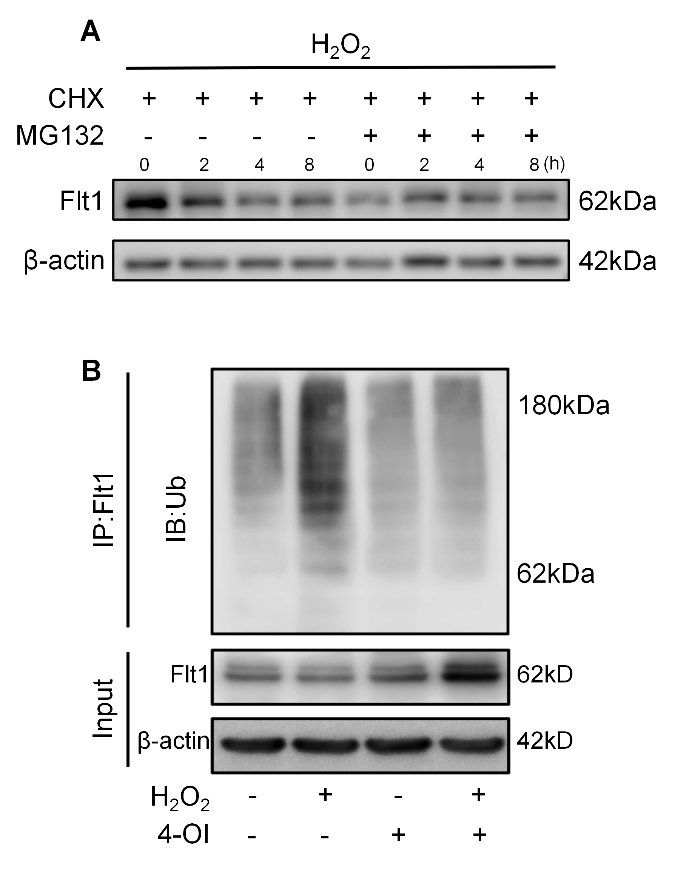
**

**Figure S7. 4-OI reduces ubiquitination levels of Flt1 in HUVECs treated with H_2_O_2_.A**, H_2_O_2_ induced the time-dependent degradation Flt1, when de novo protein synthesis was inhibited by cycloheximide (CHX) and degradation was inhibited by the proteasome inhibitor, MG132. **B**, By IP assay, 4-OI significantly reduced Flt1 ubiquitination induced by H_2_O_2_.

**Supplementary Figure 8**

**
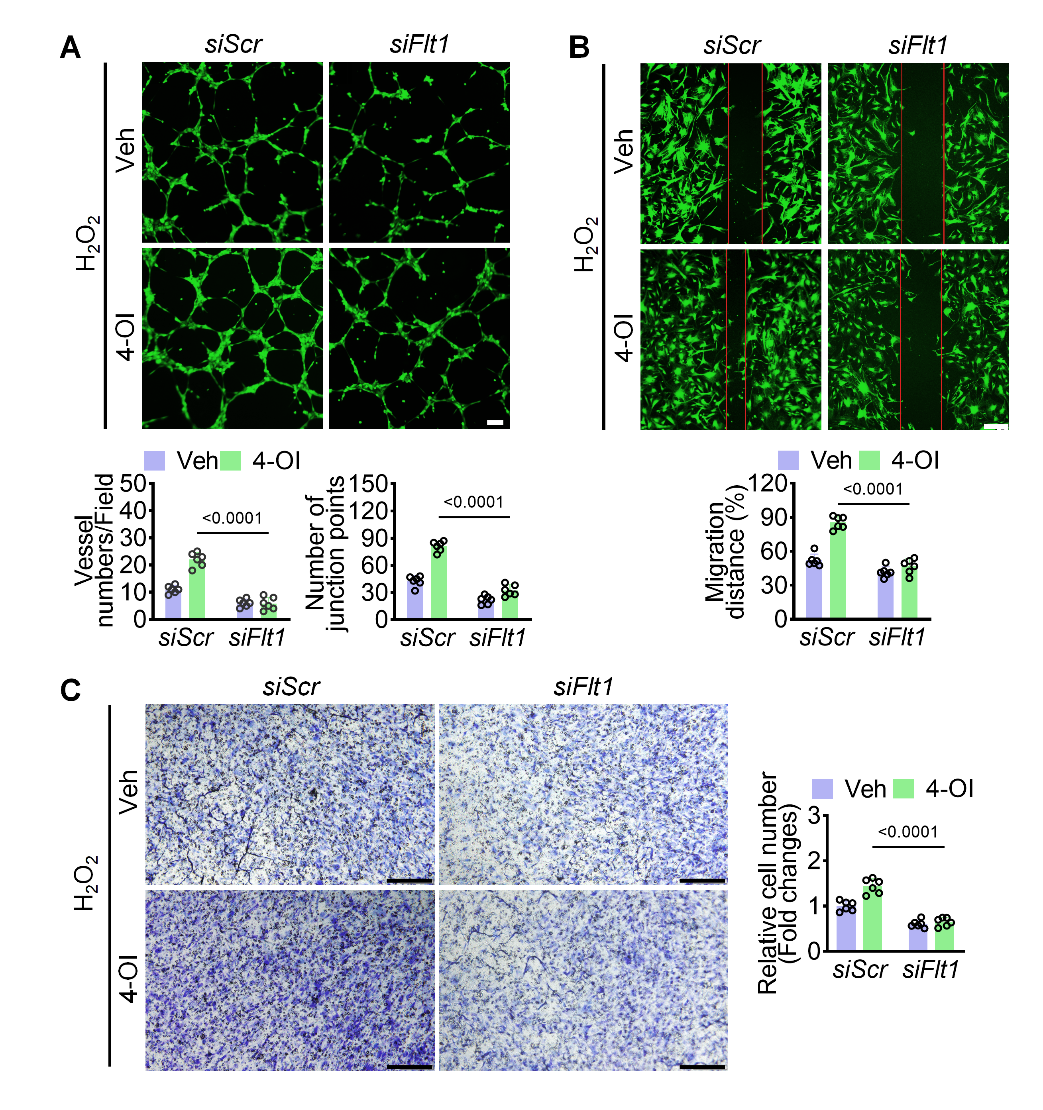
**

**Figure S8. The knockdown of Flt1 reverses the protective effect of 4-OI in HUVECs.A-C**, Under hypoxia condition, the HUVEC cells were treated with siScr or siFlt1. **A**, The number of tubes and junction points in tube formation (n=6) Scale bar = 100 μm. **B**, The migration distance of HUVECs treated with 4-OI was measured by the wound healing assay (n=6). Scale bar = 200 μm. **C**, 4-OI induced endothelial migration using the transwell migration assay (n=6). Scale bar = 100 μm. All data are presented as mean ± SD, and P-values are calculated using one-way ANOVA with Bonferroni correction.

**Supplementary Figure 9**

**
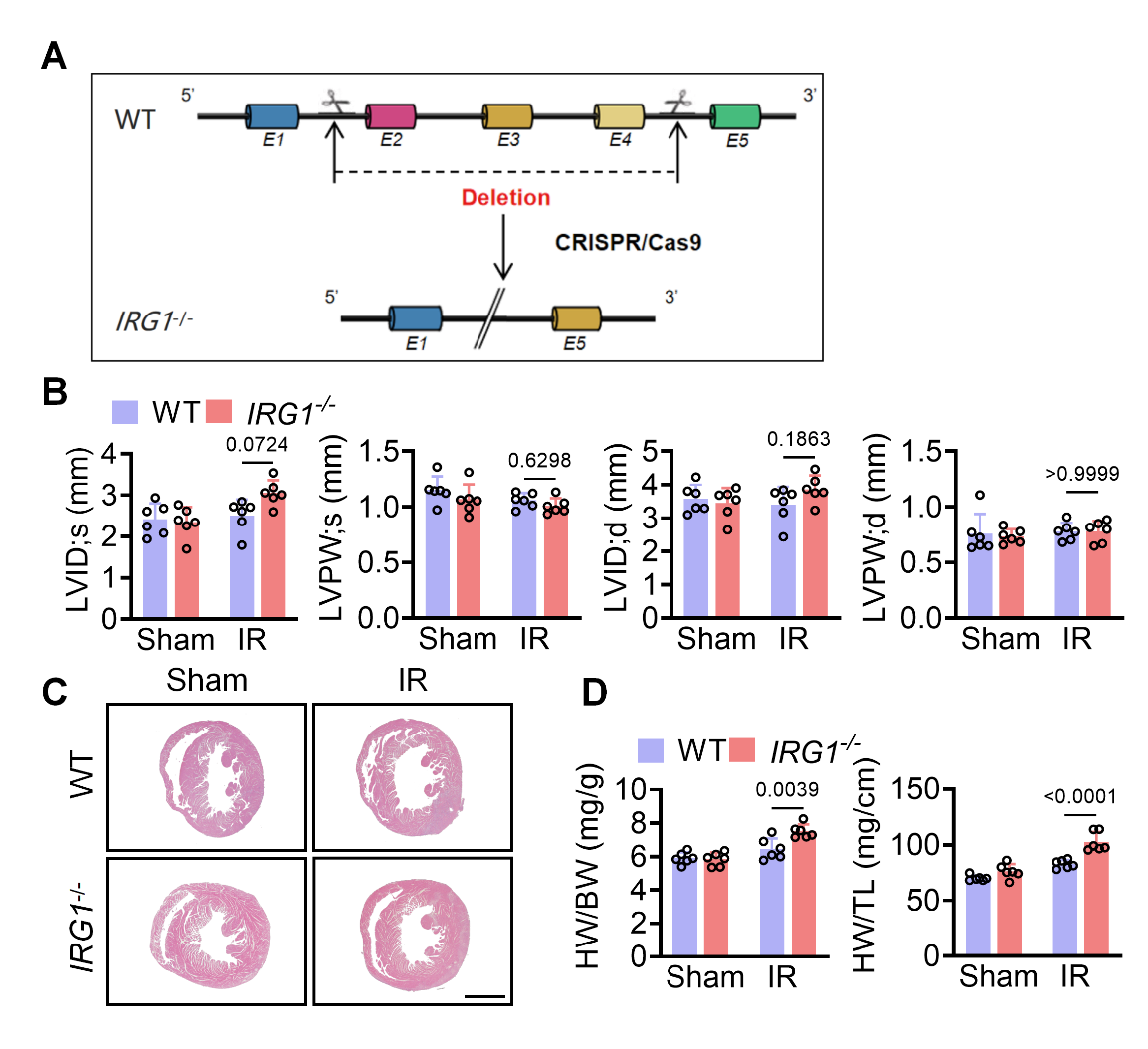
**

**Figure S9. The cardiac function changes in WT and *IRG1^-/-^*mice.A**, Strategy for the generation of global IRG1 knockout mice (*IRG1^-/-^*). **B**, 8-week-old C57BL/6 (WT) and *IRG1^-/-^* mice (n=6) were subjected to IR injury, and then echocardiography was performed. **C**, Representative hematoxylin-eosin (H&E) staining from heart tissues. Scale bar = 2 mm. **D**, The ratios for heart weight/body weight (HW/BW, mg/g) and heart weight/tibia length (HW/TL, mg/cm) post sham or IR surgery were shown (n=6). All data are presented as mean ± SD, and P-values are calculated using one-way ANOVA with Bonferroni correction.

**Supplementary Figure 10**

**
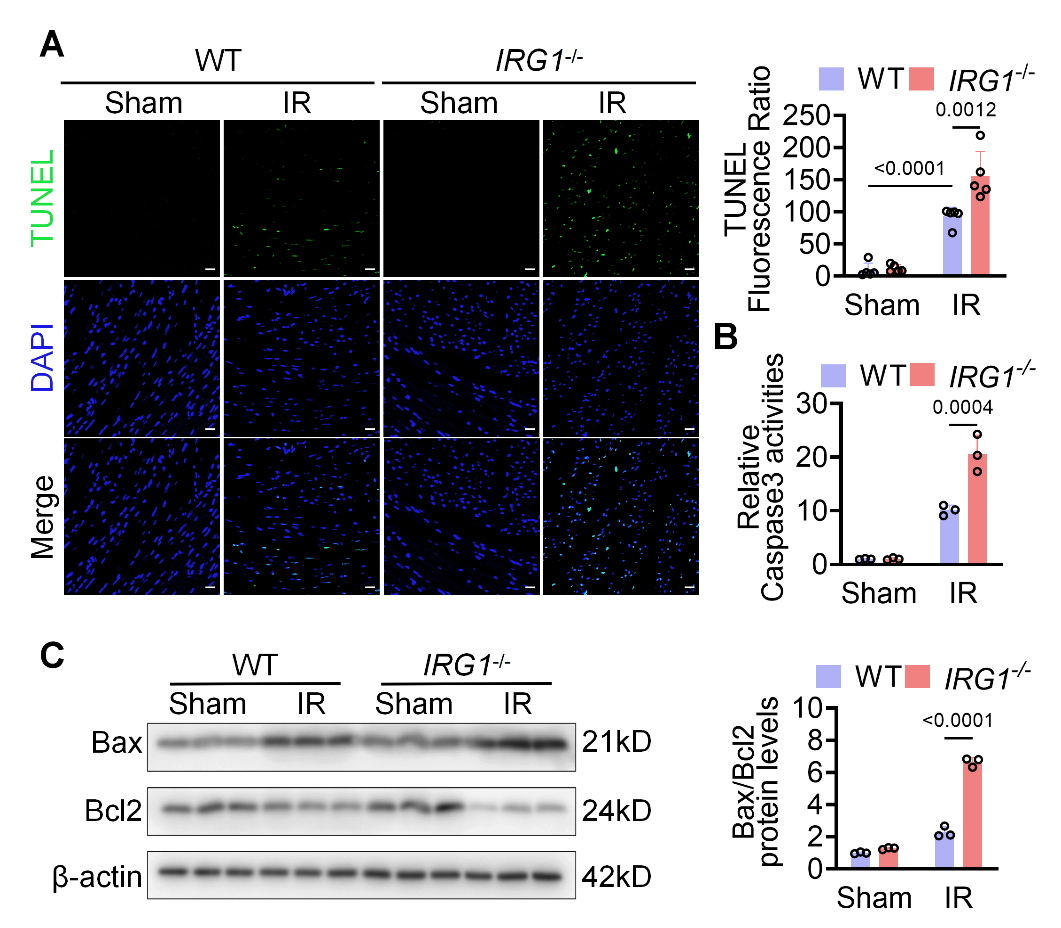
**

**Figure S10. The apoptosis level of cardiac tissue in WT and *IRG1^-/-^* mice during IR injury.A**, Representative images of TUNEL (green) staining in heart tissue (n=5). Scale bar = 20 μm. **B**, The relative Caspase 3 activities in hearts from different groups were detected (n=3). **C**, The expression of Bax and Bcl2 at protein levels was detected in heart tissues (n=3). All data are presented as mean ± SD, and P-values are calculated using one-way ANOVA with Bonferroni correction.

**Supplementary Figure 11**

**
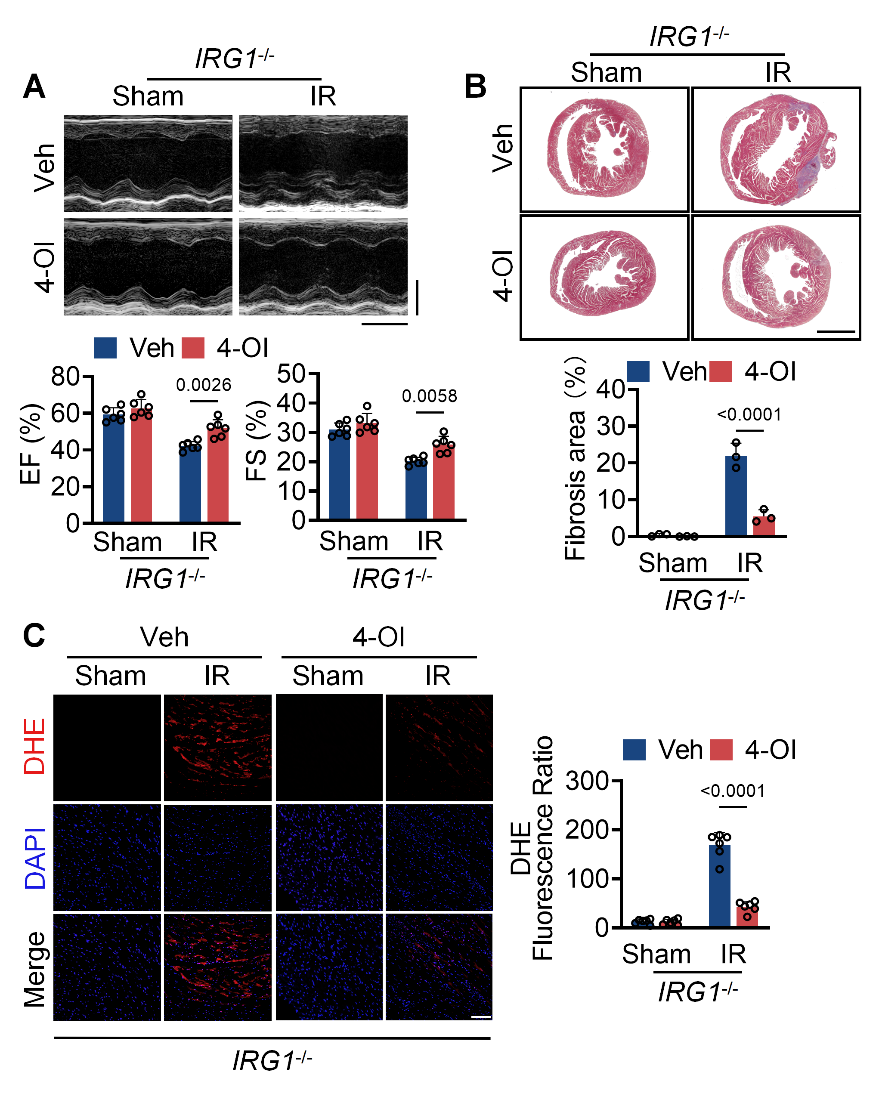
**

**Figure S11. 4-O alleviatesl heart dysfunction in *IRG1^-/-^* mice after IR injury. A**, Representative echocardiograms of mice from different groups, and quantification of left ventricular ejection fraction (EF, %), left ventricular fractional shortening (FS, %). **B**, Representative Masson trichrome staining from heart tissues (n=3). Scale bar = 2 mm. **C**, Representative images of DHE (red) staining in heart tissue (n=6). Scale bar = 20 μm. All data are presented as mean ± SD, and P-values are calculated using one-way ANOVA with Bonferroni correction.

**Supplementary Figure 12**

**
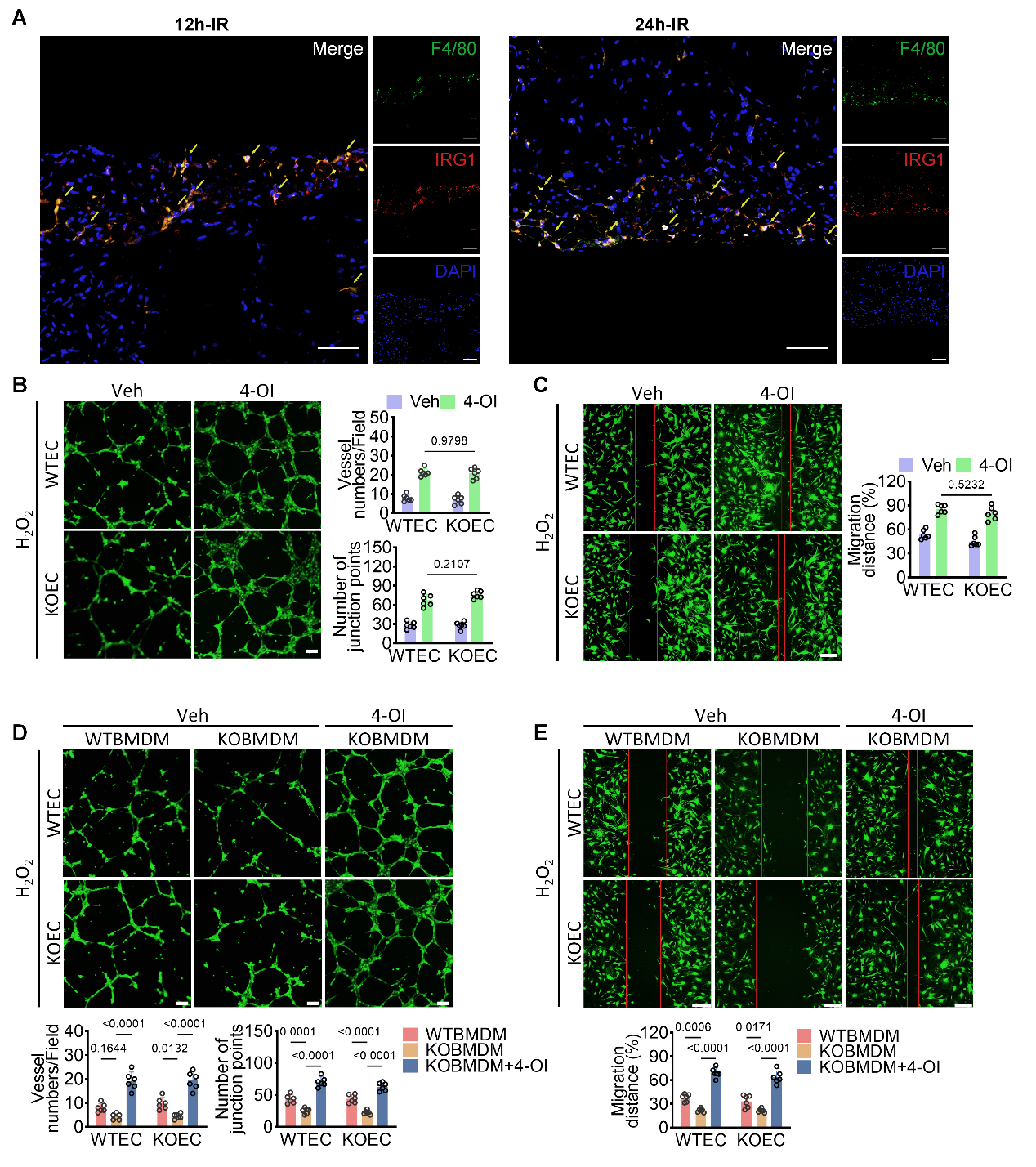
**

**Figure S12. Interaction between macrophage-derived IRG1 and endothelial cells. A**, Immunofluorescent staining was performed with antibodies against F4/80 (green) and IRG1 (red) (n=3). Scale bar = 20 μm. **B**, The number of tubes and junction points in tube formation (n=6) Scale bar = 100 μm. **C**, The migration distance of HUVECs treated with 4-OI was measured by the wound healing assay (n=6). Scale bar = 200 μm. **D**, The number of tubes and junction points in tube formation (n=6) Scale bar = 100 μm. **E**, The migration distance of HUVECs treated with 4-OI was measured by the wound healing assay (n=6). Scale bar = 200 μm. All data are presented as mean ± SD, and P-values are calculated using one-way ANOVA with Bonferroni correction.

**Supplementary Figure 13**

**
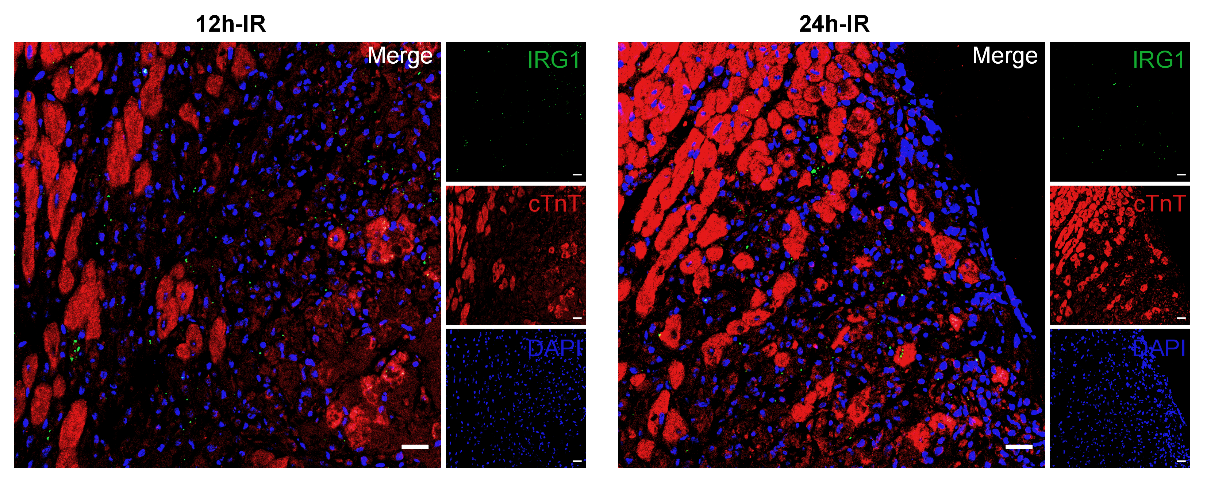
**

**Figure S13. Localization of IRG1 during early reperfusion.**

Immunofluorescent staining was performed with antibodies against IRG1 (green) and cTnT (red) (n=3). Scale bar = 20 μm.


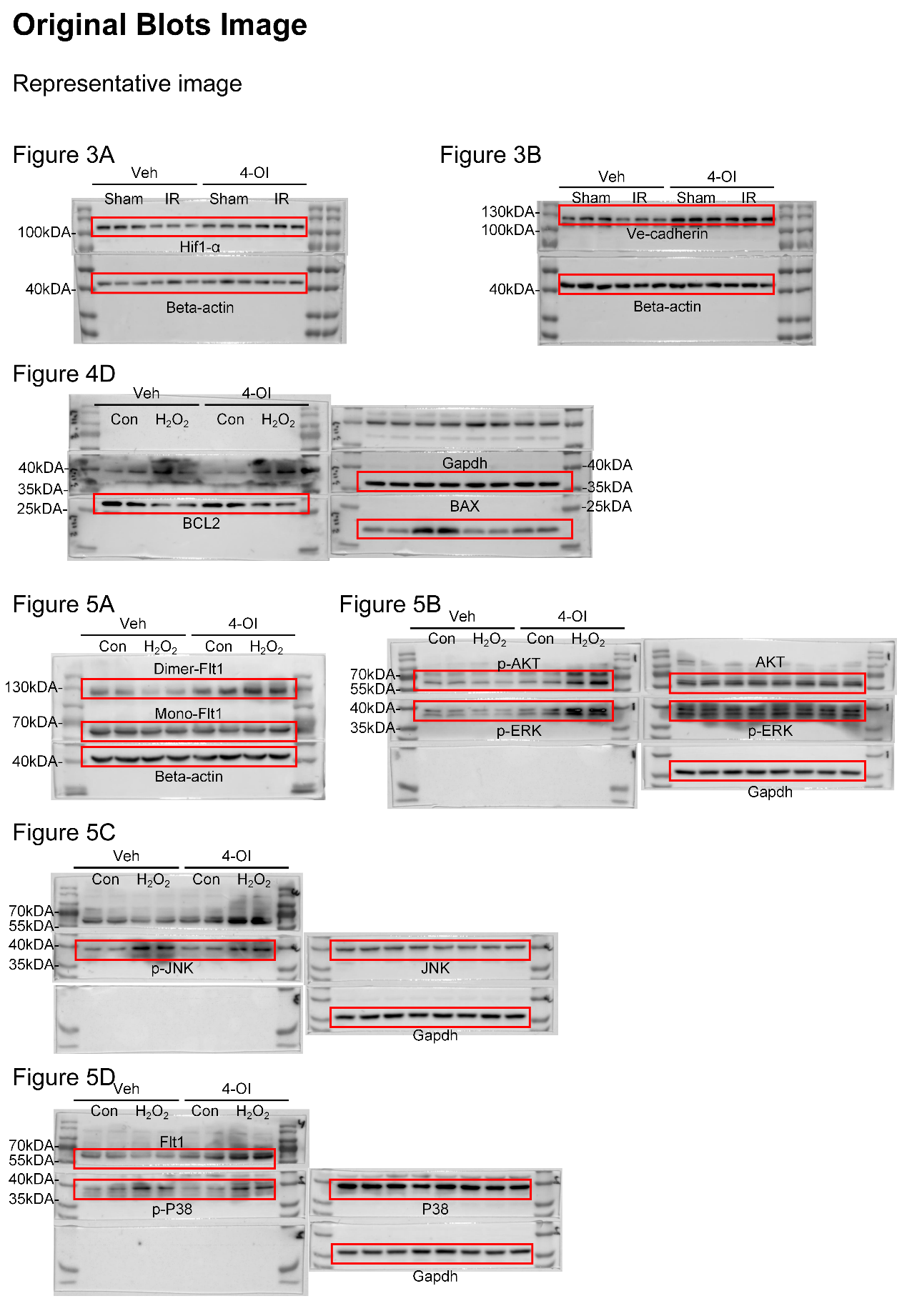


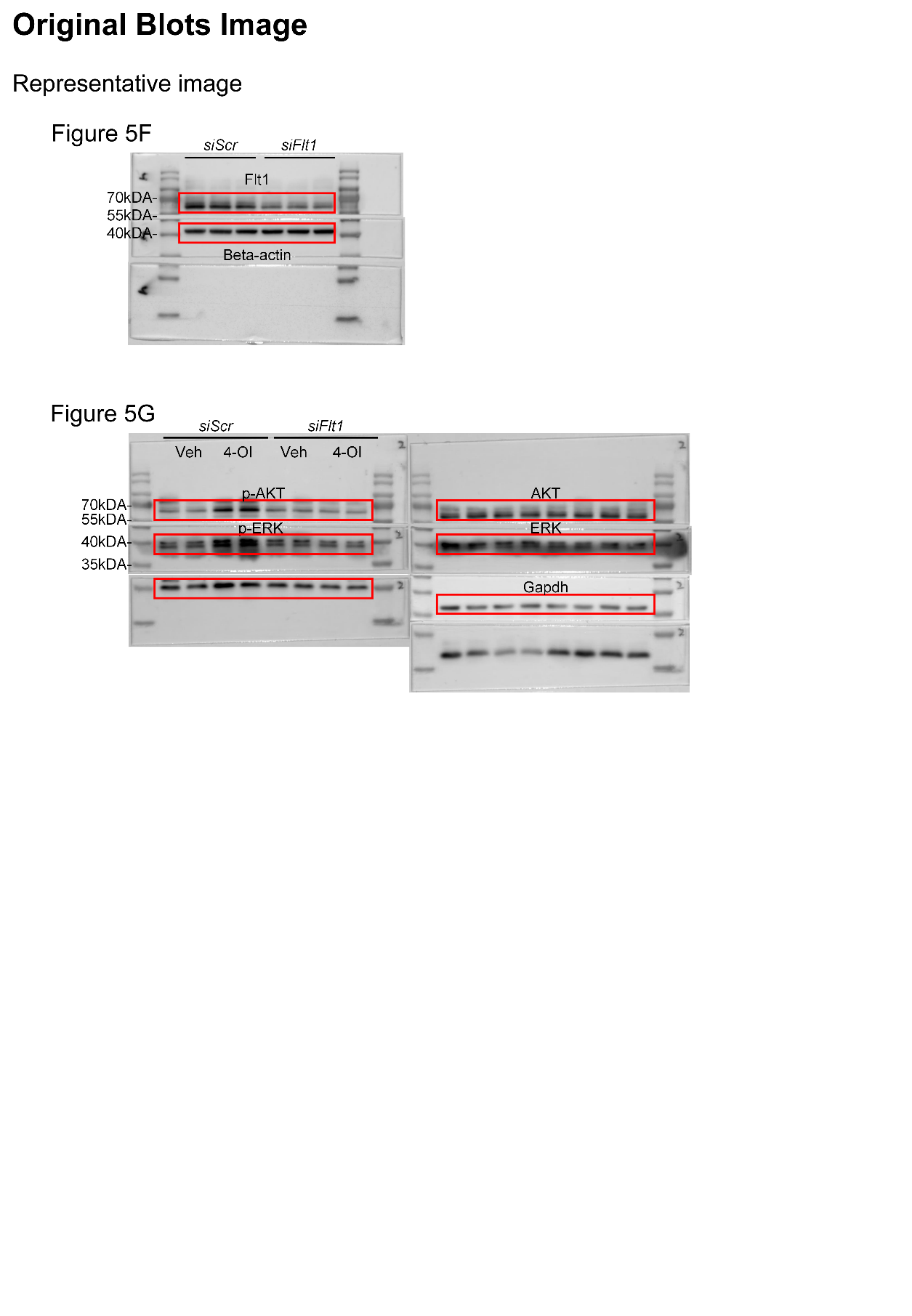


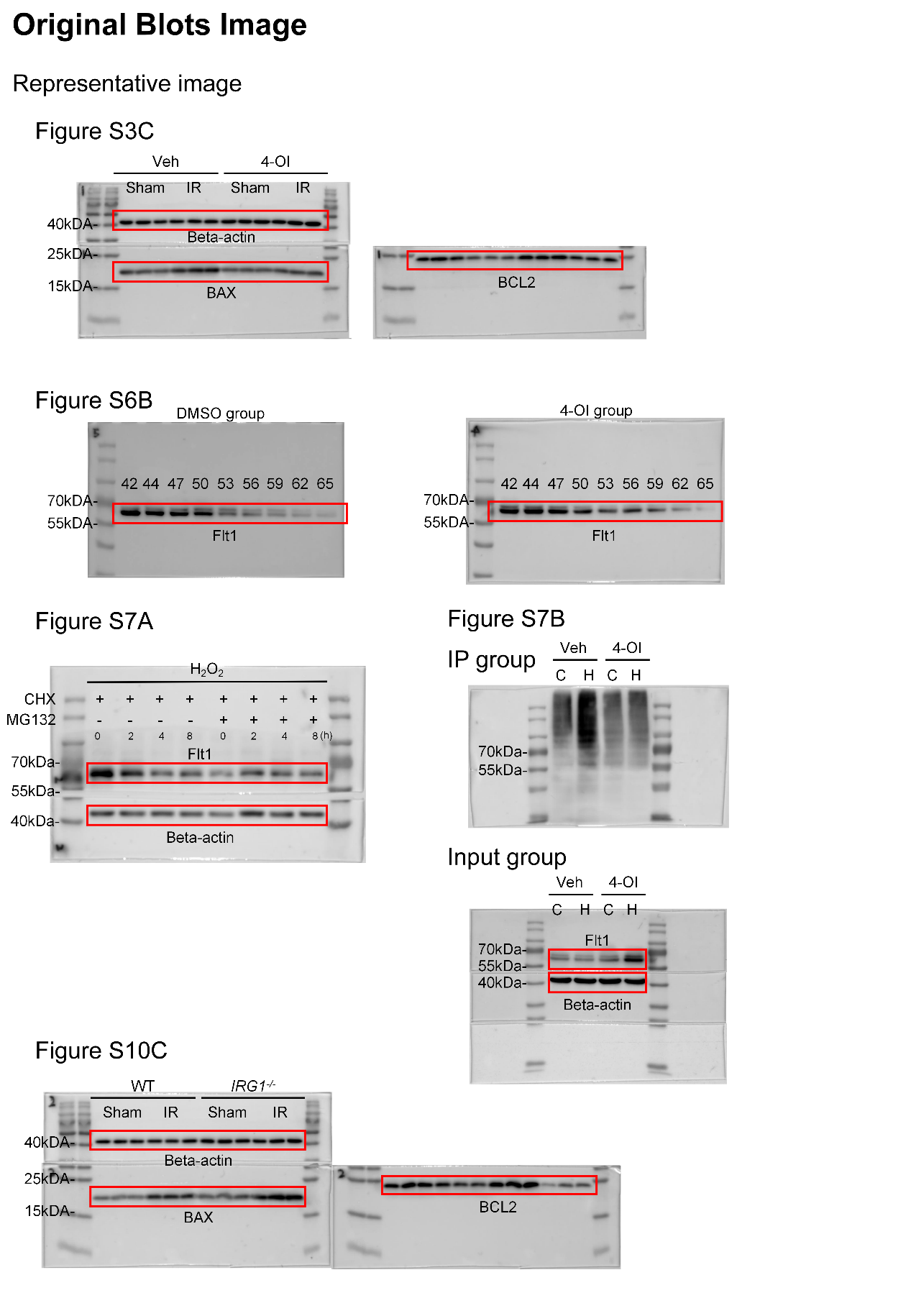

Supplement: Supplementary file 1 — Supporting Information [file ADVS-12-2411554-s001.docx]
